# Phylogeny and fatty acid profiles of new *Coccomyxa* (Chlorophyta) species from soils of Vietnam

**DOI:** 10.3389/fmicb.2025.1517865

**Published:** 2025-07-14

**Authors:** Yevhen Maltsev, Elena Kezlya, Svetlana Maltseva, Zinaida Krivova, Cù Nguyên Ðinh, Maxim Kulikovskiy

**Affiliations:** ^1^K.A. Timiryazev Institute of Plant Physiology RAS, IPP RAS, Moscow, Russia; ^2^Southern Branch of Joint Vietnam-Russian Tropical Science and Technology Research Center, Ho Chi Minh City, Vietnam

**Keywords:** Cát Tiên National Park, green algae, ITS secondary structure, new species, phylogeny, 18S rDNA

## Abstract

**Introduction:**

A new algal species of *Coccomyxa*, *C. cattiensis* sp. nov., *C. fusiformis* sp. nov., and *C. tropica* sp. nov. are recorded in the tropical forest soil from the Cát Tiên National Park, Vietnam.

**Methods:**

The analysis is based on morphological characters, evolutionary distance, 18S rDNA phylogeny, and ITS2 secondary structure.

**Results:**

New species differed from other species of the genus by the size and shape of vegetative cells, and habitat type. The evolutionary distance matrix based on the 18S rRNA gene shared 97.9–100% similarities with other *Coccomyxa* sequences. The phylogeny inferred by maximum likelihood and Bayesian inference placed new species in the independent lineages close to clades *C. subellipsoidea*, *C. polymorpha*, and *C. parasitica*. Predicted secondary structures of the ITS2 of new species differed from the *Coccomyxa* species by compensatory base changes, deletions, and single bases. Fatty acid composition analysis was performed on *Coccomyxa* strains from tropical habitats for the first time. α-linolenic (26.8–47.0%), palmitic (13.6–31.2%), and linoleic (up to 21.7%) acids were the dominant fatty acids in the algae cultured on the BBM medium.

**Conclusion:**

High concentrations of omega-3 polyunsaturated fatty acids (up to 485 mg L^–1^) position the novel *Coccomyxa* strains as a promising feedstock for the food and pharmaceutical, agricultural, and aquaculture industries.

## 1 Introduction

The diversity of soil microalgae remains poorly understood despite the widespread use of molecular methods in their study (Lawley et al., 2004; Bates et al., 2013; Hodač, 2016). One of the most extensive studies on the molecular diversity of soil green algae by culture was based on Hodač (2016) in Germany. This work selected 188 monoclonal cultures of green microalgae from 57 sample areas covering deciduous and coniferous forests, meadows, and pastures. Based on an analysis of 18S rDNA, the author distinguished 73 different phylotypes of soil green algae. The species belong mainly to the green algae: Chlorophyta (59 spp.) and Streptophyta (2 spp.), also to Stramenopiles: Xanthophyceae (11 spp.) and Eustigmatophyceae (1 sp.). We compared results obtained with a culture-dependent approach (morphological observations using light microscopy of mixed or monoclonal algal cultures) with those obtained with a culture-independent approach, NGS method based on the 18S rDNA marker (Kezlya et al., 2023). Almost 90% of cultivated species were shown to correspond to clones obtained from environmental samples, with an 18S rDNA similarity threshold of ≤ 0.01. The high proportion of matches confirms the relevance of monoclonal algal cultures studying for replenishing algal collections and amplifying other markers besides 18S rDNA. Seven new species have also been described, and it has been suggested that algae diversity in Central Europe’s soils cannot be fully explored without cultivation (Hodač, 2016).

Tropics are unique regions with high richness of eukaryotic algae and cyanobacteria. The studies of the last two decades have mainly focused on the composition and dynamics of species diversity in cultivated soils or rice fields in India (Vijayan and Ray, 2015), Egypt (Hameed, 2006), and Laos (Fujita and Ohtsuka, 2005). However, these works were carried out without using molecular approaches. The most common direction in tropical areas is the study of aerophyte algae using molecular methods. Many new genera and species of green algae have been described precisely from subaerial habitats: *Kalinella* (Neustupa et al., 2009) from the bark of *Gigantochloa* sp. in a park in Singapore, *Hylodesmus* from decaying bare wood in a tropical forest in Singapore (Eliaš et al., 2010), *Xylochloris* from tropical trees in Singapore (Neustupa et al., 2011), *Elliptochloris bilobata* var. *corticola* from Cibodas Botanical Garden West Java in Indonesia (Eliáš et al., 2008), *Symbiochloris tropica* from the bark sample in the secondary lowland rain forest in Malaysia (Škaloud et al., 2016). Data on the phylogeny and distribution of green aerophytic algae in the tropical rainforest in Yunnan, China, were also renewed, and a new order Watanabenales was proposed (Li et al., 2021). New species from the genera *Calidiella*, *Jaagichlorella*, *Massjukichlorella*, *Watanabea* were described by Li et al. (2020; 2021).

The genus *Coccomyxa* (Trebouxiophyceae, Chlorophyta) was first described by Schmidle (1901). The genus included green coccoid small size microalgae with a single parietal chloroplast lacking pyrenoids and the absence of flagellate stages and with mucilaginous sheath (Cao et al., 2018a,2018b). Several algal strains morphologically similar to *Coccomyxa* have been redefined and even described as new species. For instance, *Choricystis* sp. CAUP H 5107, *Choricystis* sp. GSE4G, *Monodus* sp. CR2-4, *Paradoxia multiseta* UTEX LB 2460, and *Pseudococcomyxa simplex* CCAP 812/5 were transferred to *Coccomyxa* based on their phylogeny (Darienko et al., 2015; Malavasi et al., 2016). The first revision of the genus using molecular methods was carried out by Darienko et al. (2015). As a result of a phylogenetic analysis of 41 strains, it was proposed to distinguish seven species. Later, Malavasi et al. (2016) performed a similar study by adding 18 more 18S rDNA + ITS sequences with the strains’ ecological features (life forms and habitats). *Coccomyxa* is widely distributed in various niches: marine, freshwater, and soil, free-living strains with epiphytic, endophytic, parasitic, and symbiotic lifestyles (Darienko et al., 2015; Malavasi et al., 2016; Gustavs et al., 2017; Cao et al., 2018b; Sciuto et al., 2019). Some species are found in extreme habitats: *C*. *onubensis* and *C*. *silvae-gabretae* in acidic waters (Garbayo et al., 2012; Fuentes et al., 2016; Barcytë and Nedbalová, 2017), *C*. *melkonianii* in the river with high content of heavy metals (Malavasi et al., 2016). The following species have been recorded in the tropical region: *C*. *actinabiotis*, strain KN-2011-T4 on the *Bambussa* sp., *C*. *polymorpha*, strain KN-2011-T2 on the bark of *Ehretia javanica*, *C*. *solorinae*, strain KN-2011-T5 on the tree bark in Singapore, strain *Coccomyxa* sp. KN-2011-T1 on the tree bark in Indonesia, strain *Coccomyxa* sp. KN-2011-T3 on the bark of *Syzygium nervosum* (Malavasi et al., 2016). *Coccomyxa* was found in Cuba as a symbiont of lichen-forming fungi of the genus *Sticta* (Ascomycota, Peltigeraceae) (Lindgren et al., 2020).

Coccoid green algae are one of the promising sources of metabolites that can be utilized for bioremediation and biomass production, with subsequent processing into biofuel, fertilizer, or feed (Lamminen et al., 2019; Ma et al., 2019; Maltsev and Maltseva, 2021). A group with a C16–C18 chain length usually dominates green algae’s fatty acid profiles. The most common fatty acids (FAs) are 16:0 palmitic, 16:3n-3 roughanic, 18:1n-9 oleic, 18:3n-3 α-linolenic acids (Lang et al., 2011; Maltsev and Maltseva, 2021). Some green algae can produce large amounts of unsaturated fatty acids (UFA), which is more than 97.0% of the total fatty acid content (Øezanka et al., 2008; Lang et al., 2011). The maximum values are set for *Chlamydomonas*, *Chloromonas* and *Choricystis* strains. Only in some *Chlamydomonas* and *Didymogenes* strains is the omega-3 fatty acid content higher than 80% (Maltsev and Maltseva, 2021). At the same time, *Coccomyxa* strains represent a source of different fatty acid groups, which can reach high concentrations depending on culture conditions. For example, saturated fatty acids (SFAs) marked in quantity up to 45.50% of total FA content (Soru et al., 2019), monounsaturated fatty acids (MUFAs) with 60.09% (Maltsev et al., 2019), polyunsaturated fatty acids (PUFAs) with 75.30% or omega-3 FAs with 56.39% (Lang et al., 2011). The high content of PUFAs and omega-3 FAs, along with the ability to rapidly increase biomass in photobioreactors (Abe et al., 2014; Soru et al., 2019), enhances the biotechnological potential of *Coccomyxa* strains for agricultural feed production.

The authors first started the study of soil algae in Cát Tiên National Park using an integrative approach in 2019. Soil samples were collected from flooded and non-flooded forest areas, as disturbed and undisturbed fields. Previous papers present new species of the different groups of algae: diatoms (Kezlya et al., 2020; Kezlya et al., 2021; Glushchenko et al., 2022; Kezlya et al., 2022), as well as cryptophytes (Martynenko et al., 2022) and cyanobacteria (Maltseva et al., 2022). During a study of the tropical soils of the Cát Tiên National Park, we isolated five strains with a *Coccomyxa*-like morphology but with several unique morphological and phylogenetic characteristics. Thus, we describe three new species from the genus *Coccomyxa* based on morphology, 18S rDNA phylogeny, ITS2 secondary structures and fatty acid profiles.

## 2 Materials and methods

### 2.1 Isolation and cultivation

**FIGURE 1:**
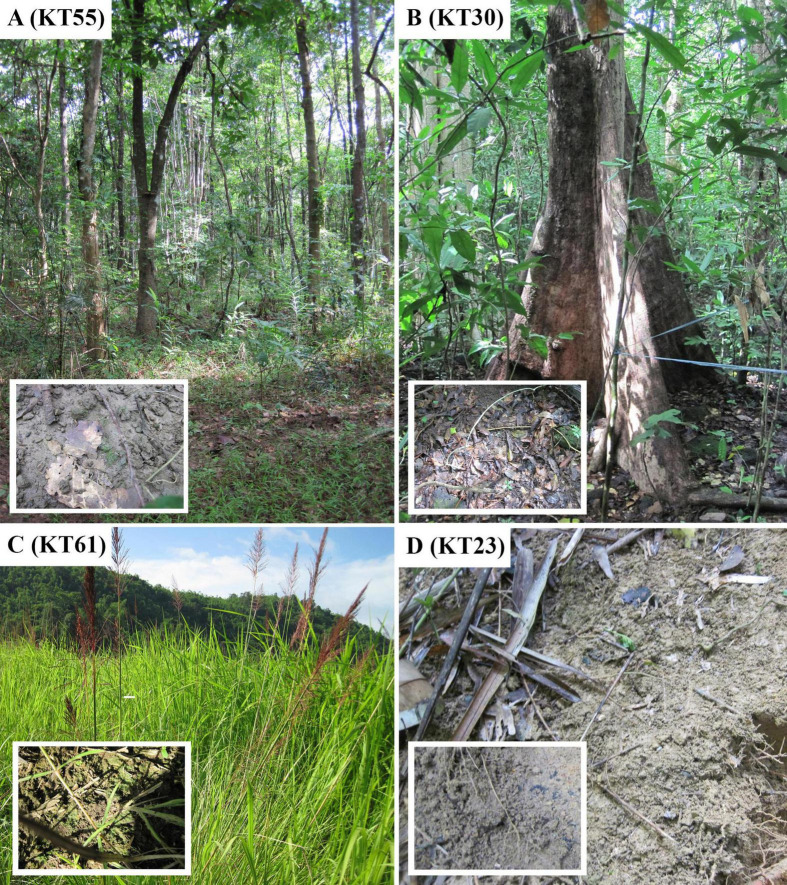
Sampling sites in Cát Tiên National Park, Đồng Nai Province, Vietnam. **(A)** Forest (KT55); **(B)** forest (KT30); **(C)** dry swamp (KT61); **(D)** forest (KT23).

Tropical forest soil samples (Figure 1) were collected in the Cát Tiên National Park, Vietnam, which has a monsoon tropical climate with summer rains. Table 1 presents a list of all samples, along with their geographic locations and measured ecological parameters.

**TABLE 1:** List of sampling locations with descriptive information.

Sample name	Geographic coordinates, local name	Habitat type	pH	Humidity, %	Relief and vegetation
KT23*	11°26.906′ N, 107°26.490′ E “Dipterocarpus”	Forest	3.8	41.1	Top of the shale ridge. The soil is red-yellow tropical shallow underdeveloped clayey on metamorphic shale. Vegetation: *Dipterocarpus turbinatus*, *Shorea roxburghii, Swintonia floribunda*, herbaceous vegetation with a protective cover of 50%, background: *Taenitis blechnoides*. The site is sufficiently lightened. The Dipterocarpaceae leaves are dominate in litterfall.
KT30*	11°25.725′ N, 72107°25.650′ E “Lagerstroemia”	Forest	5.5	39.24	The drive-side slope was very gently sloping and flooded during the wet season. Clayey slates. *Lagerstroemia calyculata* dominates the forest stands with an admixture of *Tetrameles nudiflora*, no grass. Litter is composed mainly of the bark and leaves of *L. calyculata*. The dark clayey tropical soils developed from the derivatives of basaltic rocks.
KT55	11°23.917′ N, 107°22.523′ E	Forest	5.55	44.8	Level surface with slightly pronounced microtopography/restored forest, lightened area, no flooding during the wet season. There is no exact description of vegetation and soil features.
KT61	11°30.999′ N, 107s°23.269′ E	Dry swamp	5.11	49.02	Grassy wetland. Usually, it floods during the wet season and dries up during the dry season. There was no water at the time of sampling. There is no exact description of vegetation and soil features.

*Morphological and chemical properties, bulk elemental composition and forms of iron compounds in the Cát Tiên National Park soil plot presented in Khokhlova et al. (2017).

A brief description of vegetation and soil type for samples KT23 and KT30 is given using data from Anichkin (2011) and Khokhlova et al. (2017). The authors describe the samples KT55 and KT61. Immediately after sampling, the absolute humidity was determined by the “hot drying” method (Vadjunina and Korchagina, 1986) in the laboratory room, then brought to the air-dry state and packaged. To measure pH, 30 g of soil was weighed, to which 150 mL of distilled water was added (Arinushkina, 1970). The suspension was poured into a clean glass and measured using the Hanna HI 98112 Piccolo 2 (Hanna Instruments, Inc., Woonsocket, RI, United States).

The novel strains VP336, VP339, VP449, VP451, and VP521 were isolated by micropipetting from algae enrichment cultures using an inverted Zeiss Axio Vert A1 microscope (Zeiss, Germany). Small amounts of soil samples were taken to obtain enrichment cultures, placed in Petri dishes, and moisturized. The strains were deposited in the Culture and Barcode Collection of Microalgae and Cyanobacteria “Algabank” (WDCM 1318) at K.A. Timiryazev Institute of Plant Physiology RAS (Moscow, Russia) as perpetually-transferred pure cultures.

Light microscopic observations were performed with a Zeiss Axio Scope A1 (Carl Zeiss Microscopy GmbH, Göttingen, Germany) microscope equipped with an oil immersion objective (Plan-apochromatic × 100/n.a.1.4, Nomarski differential interference contrast, DIC) and a Zeiss Axio Cam ERc 5s camera (Carl Zeiss NTS Ltd., Oberkochen, Germany). Cells were stained with 0.1% (w/v) methylene blue solution and 1.0% (w/v) ink solution to determine the mucilage structure. Observation of the strain lasted from 24 h to 6 months. The culture was maintained on the BBM medium (Bischoff and Bold, 1963). Starvation experiments using a 3N-BBM + V medium, as described by Darienko et al. (2015), were conducted to induce mucilage formation.

For biochemical analysis, the cultures were maintained in 250 mL Erlenmeyer glass flasks containing 150 mL of standard BBM medium under constant orbital shaking (150 rpm in ELMI Sky Line Shaker S-3 L, ELMI Ltd., Riga, Latvia) for 15 days. The cultures grew at standardized conditions with 24°C under light intensity 70 μmol photons m^–2^ s^–1^ with color temperature 4,000 K and a 16:8 h light/dark photoperiod. The light intensity and color temperature were measured using the Sekonic C-800 spectrometer (Sekonic Corporation, Tokyo, Japan). During strain growth, biomass increase was monitored by optical density changes using IMPLEN Nanophotometer P300 (Implen GmbH, Germany) at λ = 720 nm (OD_720_).

### 2.2 Molecular analysis

The DNA of the investigated strains VP336, VP339, VP449, VP451, and VP521 were extracted using Chelex 100 Chelating Resin, molecular biology grade (Bio-Rad Laboratories, Hercules, CA, USA), according to the manufacturer’s protocol (Bio-Rad, 2020). Amplification of the 18S rRNA gene (1,741–1,791 bp) was performed with a pair of primers 18S–FA2 (5′–ACC TGG TTG ATC CTG CCA GTA–3′) and 18S–RB2 (5′–GAT CCT TCT GCA GGT TCA CCT ACG–3′) (Nakada et al., 2010). Amplification conditions for the 18S rRNA gene were as follows: initial denaturation for 5 min at 95°C followed by 32 cycles of 30 s denaturation at 94°C, 40 s annealing at 64°C, and 90 s extension at 72°C, with the final extension for 5 min at 72°C. Amplification of the site of ITS1–5.8S rDNA–ITS2 region (576–744 bp) was performed with a pair of primers ITS1 (5′–TCC GTA GGT GAA CCT GCG G–3′) and ITS4 (5′–TCC TCC GCT TAT TGA TAT GC–3′) (White et al., 1990). Amplification conditions for the ITS1–5.8S rDNA–ITS2 region were as follows: initial denaturation for 5 min at 95°C followed by 35 cycles of 30 s denaturation at 94°C, 30 s annealing at 60°C, and 60 s extension at 72°C, with the final extension for 5 min at 72°C.

The PCR products were visualized by horizontal electrophoresis in 1.0% agarose gel stained with SYBR™ Safe (Life Technologies, Carlsbad, CA, United States). The products were purified with a mixture of FastAP, 10 × FastAP Buffer, Exonuclease I (Thermo Fisher Scientific, Waltham, MA, United States), and water. Sequencing was performed using a Genetic Analyzer 3500 instrument (Applied Biosystems, Waltham, MA, United States). We used internal primers provided by Maltsev et al. (2018) for sequencing.

**FIGURE 2:**
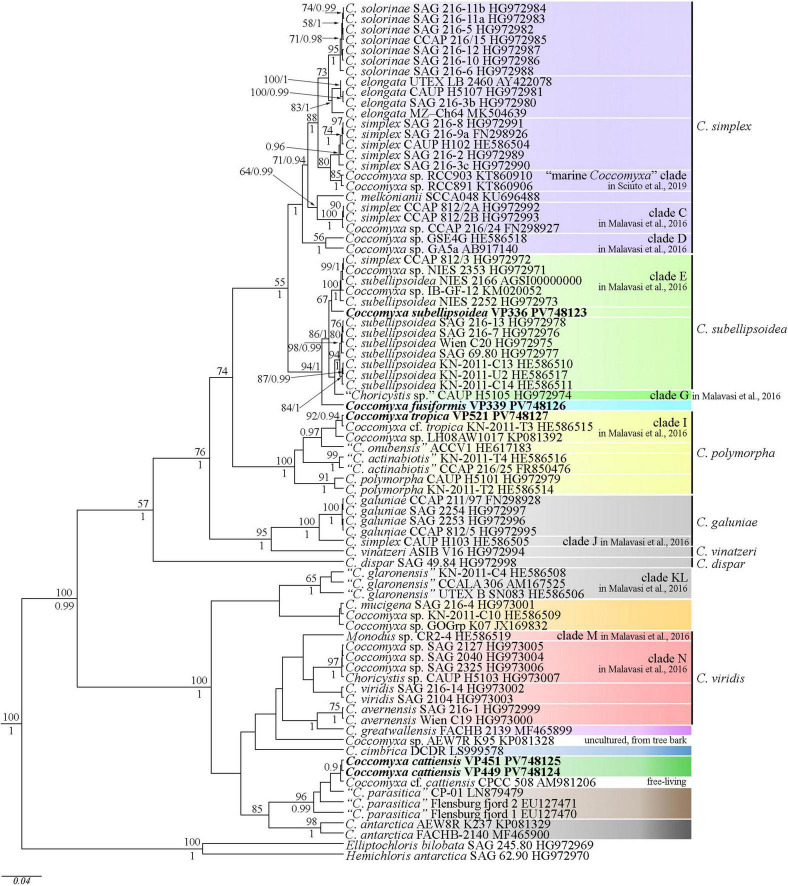
Phylogenetic position of new *Coccomyxa* species (indicated in bold) based on Bayesian inference from an alignment of 82 sequences and 2,706 characters (18S rRNA gene and ITS1–5.8S rDNA–ITS2 region). Values above the horizontal lines are bootstrap support from ML analyses (values below 50 are not shown). Values under the horizontal lines (or to the right of the slash) are Bayesian posterior probabilities (values below 0.9 are not shown). Strain numbers (if available) and NCBI database accession numbers are indicated for all sequences. Colored boxes represent clades according to the most recent classification or OTUs described in this work or previous papers. The classification sensu Darienko et al. (2015) marked to the right of the vertical line.

Editing and assembling of the consensus sequences were carried out by processing the direct and reverse chromatograms in Ridom TraceEdit (ver. 1.1.0) (Ridom GmbH, Münster, Germany) and Mega (ver. 7.0.26) software. The 18S rDNA and ITS1–5.8S rDNA–ITS2 sequences of the novel strains were included in the alignments of 75 *Coccomyxa* sequences from the National Center for Biotechnology Information^1^ (taxa names and Accession Numbers are given in Figure 2). *Elliptochloris bilobata* and *Hemichloris antarctica* were chosen as the outgroups. The 18S rDNA and ITS1–5.8S rRNA–ITS2 sequences of all strains (including outgroups) were aligned in Mega (ver. 7.0.26) software according to their secondary structure by means of comparing the structure presented for the strain NIES 2166 of *C*. *subellipsoidea* and SAG 216-3b of *C*. *simplex* (Darienko et al., 2015). The resulting alignments had lengths of 2,706 characters.

The data set was analyzed using the Bayesian inference (BI) method implemented in Beast ver. 1.10.1 software (BEAST Developers, Auckland, New Zealand) (Drummond and Rambaut, 2007) to construct a phylogeny. The most appropriate substitution model for the alignment partition, shape parameter α and a proportion of invariable sites (pinvar) were estimated using the Bayesian information criterion (BIC) as implemented in jModelTest ver. 2.1.10 (Vigo, Spain) (Darriba et al., 2012). This BIC-based model selection procedure selected the TrN + I + G model, shape parameter α = 0.3770 and a proportion of invariable sites (pinvar) = 0.5500.

We used the HKY model of nucleotide substitution instead of TrN, given that it was the best matching model available for BI. A Yule process tree prior was used as a speciation model. The analysis ran for 5 million generations with chain sampling every 1,000 generations. The parameters-estimated convergence, effective sample size (ESS), and burn-in period were checked using the Tracer ver. 1.7.1 software (MCMC Trace Analysis Tool, Edinburgh, United Kingdom) (Drummond and Rambaut, 2007). The initial 25% of the trees were removed, and the rest were retained to reconstruct a final phylogeny. The phylogenetic tree and posterior probabilities of its branching were obtained based on the remaining trees, having stable estimates of the parameter models of nucleotide substitutions and likelihood. The Maximum Likelihood (ML) analysis was performed using RAxML software (Stamatakis et al., 2008). The non-parametric bootstrap analysis with 1,000 replicas was used. FigTree ver. 1.4.4 (University of Edinburgh, Edinburgh, United Kingdom) and Adobe Photoshop CC ver. 19.0 software (Adobe, San Jose, CA, United States) were used for viewing and editing the trees.

The Mfold version 2.5 software was used to model the secondary structure of ITS2 (Zuker, 2003). The presence of UU pyrimidine–pyrimidine unpaired section in the second hairpin and the conservative GGUAG motive in the 5′ side of helix III (Coleman, 2007), as well as the length and nucleotide composition of the spacers in the central loop determining the helix boundaries, were taken into consideration when constructing the final ITS2 model (Caisová et al., 2013). Also, we used a concept of ITS2 secondary structure provided by Darienko et al. (2015) for *Coccomyxa* to avoid ambiguity in the interpretation of results. The construction of a hybrid stem with the 5.8S rDNA terminal site and the complementary 28S rDNA start site determined the beginning and the end of ITS2. The PseudoViewer3 program (Byun and Han, 2009) and Adobe Photoshop CC ver. 19.0 software were used to visualize the resulting secondary structure.

The 18S rRNA gene or whole 18S rDNA–ITS1–5.8S rDNA region (if available) was also used to estimate the degree of similarity between gene sequences of different *Coccomyxa* strains. Using Mega (ver. 7.0.26) software, the *p*-distances were determined to calculate the sequence similarity with the formula (1–*p*) × 100.

### 2.3 Fatty acid composition analysis

Biomass preparation for the fatty acid methyl ester (FAME) profiling was performed according to Maltseva et al. (2022). The VP336, VP339, VP449 and VP521 cultural suspensions with 150 ± 6.0 mg algal wet weight were transferred to 50 mL tubes. The cells were pelleted at room temperature for 3 min at 3,600 *g*. The supernatants were removed, and the pelleted cells were resuspended in a 10 mL volume of distilled water, quantitatively transferred to 15 mL centrifuge tubes, and pelleted again by centrifugation. The supernatants were removed, and all the quantities of biomass were transferred to a 50-mL round-bottom flask. To avoid the oxidation of unsaturated FAs, all samples were dried with an argon flow. Heptadecanoic acid (Sigma-Aldrich, United States) was used as an internal standard for determining fatty acid composition (Maltseva et al., 2022). Ten milliliter of 1M KOH in 80% aqueous ethanol were added to the dry residue, the flask was sealed with a reflux condenser and kept for 60 min at the mixture’s boiling point (∼80°C). After the time-lapse, the solvents were evaporated *in vacuo*. The resulting volume of ∼3 mL was quantitatively transferred to a 50-mL centrifuge tube with distilled water added to a total volume of 25 mL. Unsaponifiable components were extracted with three 10 mL changes of *n*-hexane (Himmed, Russia); the tube was centrifuged at room temperature for 5 min at 2,022 g to accelerate phase separation. After that, the aqueous phase was acidified with a few drops of 20% sulfuric acid (Himmed, Russia) to a slightly acidic pH (checked with indicator paper). Free FAs were extracted with 15 mL of *n*-hexane. The extraction was repeated three times. The hexane solution of free FAs was transferred to a dry 50-mL round-bottom flask, and the solvent was evaporated to dryness on a rotary evaporator (IKA RV-10, Germany), after which 10 mL of absolute methanol (Sigma-Aldrich, United States) and 1 mL of acetyl chloride (Sigma-Aldrich, United States) were added to the dry residue. The flask, closed with a reflux condenser, was kept for 1 h at 70°C, then the solvents were evaporated to dryness, a few drops of distilled water were added to the dry residue, and FAMEs were extracted with *n*-hexane.

The obtained FAMEs were analyzed using an Agilent 7890A GC gas chromatograph (Agilent Technologies, United States) equipped with an Agilent 5975Ñ mass spectrometric detector and G2591A inert ion source. A DB-23 capillary column (Ser. no. US8897617H, B&W, United States), 60 m long and 0.25 mm in diameter and containing a grafted (50% cyanopropyl) methylpolysiloxane polar liquid phase as a 0.25-mm-thick film, was used for the analysis. The remaining conditions of the analysis were as follows: pressure in the injector 191 kPa, carrier gas helium at a flow rate of 1 mL min^–1^, injected sample volume 1 μL, 1:5 flow split ratio, and evaporation temperature of 260°C. The temperature gradient program was as follows: 130–170°C at 6.5°C min^–1^ steps; 170–215°C at 2.5°C min^–1^ increments, hold at 215°C for 25 min, 215–240°C at 40°C min^–1^ increments, and the final hold at 240°C for 50 min; the operating temperature of the mass spectrometric detector was 240°C and ionization energy was 70 eV.

### 2.4 Data analysis

All analyses were performed in triplicate. Tables show the mean values and standard errors. The significance of differences between the groups was evaluated by one-way analysis of variance (ANOVA) with a Tukey’s *post-hoc* test performed using Statgraphics Centurion ver. 18 software. A difference between the two groups was declared significant at *P* < 0.05.

## 3 Results

The studies performed light microscopy, 18S rDNA phylogeny, ITS2 secondary structures, and fatty acid profiles of five new *Coccomyxa* strains. Molecular and morphological investigations showed that strains VP339, VP449, VP451, and VP521 are unknown taxa. Therefore, they are described herein as new species: *Coccomyxa fusiformis* Maltsev et Kezlya, *Coccomyxa tropica* Maltsev et Kezlya, *Coccomyxa cattiensis* Maltsev et Kezlya.

### 3.1 Description of new species and strains

#### 3.1.1 *Coccomyxa subellipsoidea* E. Acton

##### 3.1.1.1 Strain *Coccomyxa subellipsoidea* VP336

**FIGURE 3:**
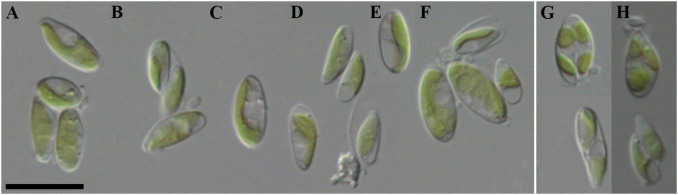
Nomarski interference micrographs of *Coccomyxa subellipsoidea*, strain VP336 in culture. Scale bar = 10 μM. **(A–F)** Variable shapes of culture cells, age 2 weeks; **(G,H)** Asexual reproduction through cell division with the formation of 2–4 autospores, age 4 weeks.

Morphological description (Figure 3): Mature vegetative cells solitary, ellipsoidal, pointed on one side, sometimes slightly curved, asymmetrical, without mucilaginous sheath, cell size 5.0–7.1 μm in length and 1.6–3.9 μm in width. Chloroplast parietal, trough-shaped, thin, straight or with small concavity in the middle, covering about half of the cell volume. Pyrenoid is absent. Cell wall is thin. Reproduction by 2–4 autospores. Protoplast division is oblique. Liberation of autospores through the rupture of mother cell wall at one end. The released autospores have the same morphology as mature vegetative cells.

Culture information: The culture was deposited and maintained as an active culture under the designation VP336 in the Culture and Barcode Collection of Microalgae and Cyanobacteria “Algabank” (WDCM 1318) at K.A. Timiryazev Institute of Plant Physiology RAS.

Habitat: Forest soil, Cát Tiên National Park, Đồng Nai Province, Vietnam (11°23.917′ N, 107°22.523′ E): soil sample KT55 collected on 25 June 2019.

Sequence data: Accession number PV748123 in the National Center for Biotechnology Information (see text footnote 1) for the 18S rRNA gene and ITS1–5.8S rDNA–ITS2 region sequence.

Remarks: In size and form of cells similar to *C. onubensis*, *C. vinatzeri*, *C. melkonianii*, *C. fottii* and *C. cattiensis*, *C. tropica* from this study (Supplementary material 1). The new strain has a thinner chloroplast, covering no more than half of the cell volume, whereas in the above species, a massive chloroplast occupies more than half of the cell volume. Also, *C. fottii, C. onubensis*, and *C. tropica* lipid droplets were present in the cytosol, whereas in *C. subellipsoidea* they were not observed.

**FIGURE 4:**
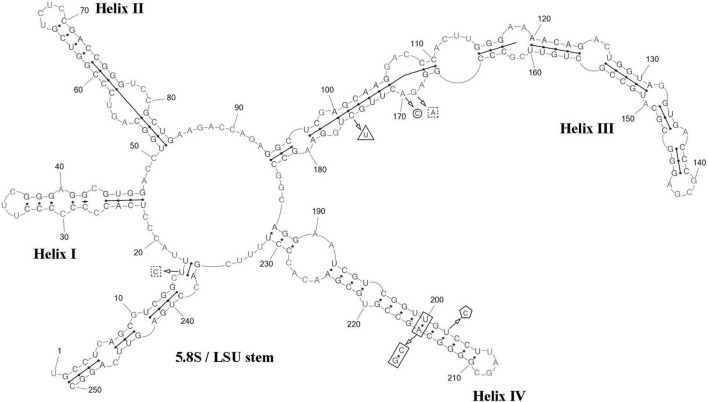
Predicted secondary structure of the ITS2 sequence of the *Coccomyxa subellipsoidea*, strain VP336. Base numbering is indicated every 10 bases. The four helices are numbered with Roman numerals. The barcode region of the ITS2 sensu Darienko et al. (2015) is shown by lines inside the secondary structure. hCBC within the barcode region for “*Choricystis* sp.” CAUP H5105 is shown by a triangle. Single bases within the barcode region for “*Choricystis* sp.” CAUP H5105 are shown by squares with dotted sides. Single base within the barcode region for *C*. *subellipsoidea* SAG 216-13, SAG 216-7 and “*Choricystis* sp.” CAUP H5105 is shown by a circle. CBC outside the barcode region for *C*. *subellipsoidea* SAG 216-13, SAG 216-7 and “*Choricystis* sp.” CAUP H5105 is shown by rectangle and hCBC is shown by pentagon. Deletions and single bases outside the barcode region are not shown.

ITS2 analysis: Comparative analysis of ITS2 sequences for the strain VP336 vs *C. subellipsoidea* SAG 216-13 (type culture), SAG 216-7 and “*Choricystis* sp.” CAUP H5105 strains revealed several evolutionary events in the clade *C. subellipsoidea* (Figures 2, 4). The VP336 and SAG 216-13, SAG 216-7 and “*Choricystis* sp.” CAUP H5105 sequences differed by one A→C transversion within the barcode region in the helix III; one compensatory base change (CBC) U–A → C–G and one hemi-compensatory base change (hCBC) U–G → C–G in the helix IV (outside the barcode region). Also, one hCBC in the helix III (G–C → G–U) and one U→C transition in the 5.5S/LSU stem differed VP336 from CAUP H5105.

#### 3.1.2 *Coccomyxa fusiformis* Maltsev et Kezlya

**FIGURE 5:**
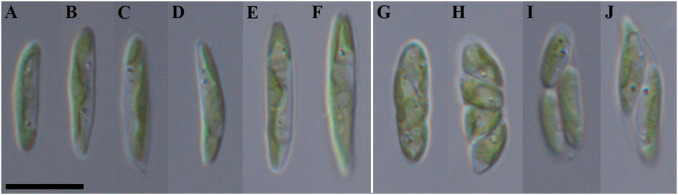
Nomarski interference micrographs of *Coccomyxa fusiformis*, strain VP339 in culture. Scale bar = 10 μM. **(A–F)** Variable shapes of culture cells, age 2 weeks; **(G–J)** asexual reproduction through cell division with the formation of 2–4 autospores, age 4 weeks.

Diagnosis (Figure 5): Mature vegetative cells solitary, elongated-oval, very often pointed on both sides, spindle-shaped, slightly curved, asymmetric, without mucilaginous sheath, cell size 8.0–15.0 (16.2) μm in length and 1.9–3.0 (3.4) μm in width. Chloroplast parietal, thin, trough-shaped or ribbon-like, with waved margin in large cells, covering about half of the cell volume. Pyrenoid is absent. Lipid droplets present in the cytosol. Cell wall is thin. Reproduction by 2–4 autospores. Protoplast division is oblique. Liberation of autospores through the rupture of mother cell wall at one end. The released autospores have the same morphology as mature vegetative cells.

Holotype (designated here): Material from the authentic strain VP339 is stored in the IPPAS collection of microalgae and cyanobacteria (WDCM 596), Moscow, Russian Federation (metabolically inactive cryopreserved culture). Preserved specimen with unfixed, dried cells (in a metabolically inactive state) from a large drop of a batch of strain VP339 added to watercolor paper (Figure 5), deposited at the MHA (Herbarium, Main Botanical Garden, Botanicheskaya Str. 4, Moscow, 127276, Russia) under the designation *Coccomyxa fusiformis* Vietnam Kezlya strain VP339. The culture was also deposited and maintained as an active culture under the designation VP339 in the Culture and Barcode Collection of Microalgae and Cyanobacteria “Algabank” (WDCM 1318) at K.A. Timiryazev Institute of Plant Physiology RAS. Also, the strain is conserved as a formaldehyde-fixed sample.

Reference strain: VP339.

Type locality: Forest soil, Cát Tiên National Park, Đồng Nai Province, Vietnam (11°30.999′ N, 107°23.269′ E): soil sample KT61 from dry swamp collected on 25 June 2019.

Sequence data: Accession number PV748126 in the National Center for Biotechnology Information (see text footnote 1) for the 18S rRNA gene and ITS1–5.8S rDNA–ITS2 region sequence.

Etymology: The species epithet “fusiformis” refers to the spindle-like cell shape.

Remarks: The new species differs in cell size and shape from both those studied in this study and the phylogenetically closely *C. subellipsoidea* (Darienko et al., 2015) as it has relatively large elongated cells (8.0–16.2 μm in length, 1.9–3.4 μm in width, Supplementary material 1). Morphologically similar to *C*. *polymorpha* (8.0–13.0 μm in length, 2.0–3.5 μm in width) but cells not gathered in star-like structures. Exact identification is possible only by using 18S rDNA and ITS2 sequence analysis.

Distribution: As yet known only from the type locality.

**FIGURE 6:**
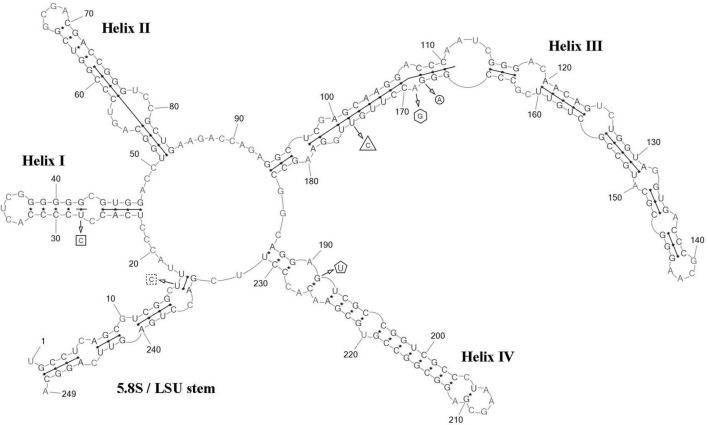
Predicted secondary structure of the ITS2 sequence of the *Coccomyxa fusiformis*, strain VP339. Base numbering is indicated every 10 bases. The four helices are numbered with Roman numerals. The barcode region of the ITS2 sensu Darienko et al. (2015) is shown by lines inside the secondary structure. hCBC within the barcode region for *C*. *subellipsoidea* SAG 216-13, SAG 216-7 and “*Choricystis* sp.” CAUP H5105 is shown by a rectangle. hCBC within the barcode region for *C*. *subellipsoidea* SAG 216-13, SAG 216-7 is shown by a triangle. Single base within the barcode region for *C*. *subellipsoidea* SAG 216-13, SAG 216-7 and “*Choricystis* sp.” CAUP H5105 is shown by a circle. A single base within the barcode region for *C*. *subellipsoidea* SAG 216-13, and SAG 216-7 is shown by hexagon. Single base within the barcode region for “*Choricystis* sp.” CAUP H5105 is shown by a square with dotted sides. hCBC outside the barcode region for *C*. *subellipsoidea* SAG 216-13, SAG 216-7 and “*Choricystis* sp.” CAUP H5105 is shown by the pentagon. Deletions and single bases outside the barcode region are not shown.

ITS2 analysis: Several modifications within the barcode region have been shown in the result of the comparison of the secondary structure of ITS2 between *C*. *fusiformis vs C. subellipsoidea* SAG 216-13, SAG 216-7 and “*Choricystis* sp.” CAUP H5105: one hCBC in the helix I (U–G → C–G) and one G→A transition in the helix III (Figure 6). Additionally, one hCBC (G–U → G–C) and one G→A transition in helix III distinguished VP339 from SAG 216-13 and SAG 216-7. One hCBC (G–C → U–C) in the helix IV (outside the barcode region) differed *C*. *fusiformis* VP339 from other taxa in the clade *C. subellipsoidea* (Figure 2). Thus, the most significant differences in nucleotide sequences of ITS2 of the strain VP339 were located inside and outside the barcode region.

#### 3.1.3 *Coccomyxa tropica* Maltsev et Kezlya

**FIGURE 7:**
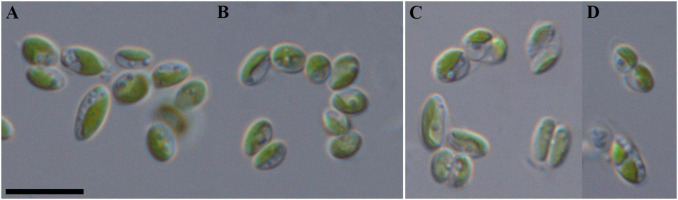
Nomarski interference micrographs of *Coccomyxa tropica*, strain VP521 in culture. Scale bar = 10 μM. **(A,B)** Variable shapes of culture cells, age 2 weeks; **(C,D)** asexual reproduction through cell division with the formation of 2–4 autospores, age 4 weeks.

Diagnosis (Figure 7): Mature vegetative cells solitary, ellipsoidal, sometimes ovoid, without mucilaginous sheath, cell size 3.3–6.9 μm in length and 1.5–2.7 μm in width. Chloroplast parietal, trough-shaped, thick, covering about half of the cell volume. Pyrenoid is absent. Lipid droplets present in cytosol. Cell wall is thin. Reproduction by 2–4 autospores. Protoplast division is oblique, rarely transverse. Liberation of autospores through the rupture of mother cell wall at one end. The released autospores have the same morphology as mature vegetative cells.

Holotype (designated here): Material from the authentic strain VP521 is stored in the IPPAS collection of microalgae and cyanobacteria (WDCM 596), Moscow, Russian Federation (metabolically inactive cryopreserved culture). Preserved specimen with unfixed, dried cells (in a metabolically inactive state) from a large drop of a batch of strain VP521 added to watercolor paper (Figure 7), deposited at the MHA (Herbarium, Main Botanical Garden, Botanicheskaya Str. 4, Moscow, 127276, Russia) under the designation *Coccomyxa tropica* Vietnam Kezlya strain VP521. The culture was also deposited and maintained as an active culture under the designation VP521 in the Culture and Barcode Collection of Microalgae and Cyanobacteria “Algabank” (WDCM 1318) at K.A. Timiryazev Institute of Plant Physiology RAS. Also, the strain is conserved as a formaldehyde-fixed sample.

Reference strain: VP521.

Type locality: Forest soil, Cát Tiên National Park, Đồng Nai Province, Vietnam (11°26.906′ N, 107°26.490′ E): soil sample KT23 collected on 05 June 2019.

Sequence data: Accession number PV748127 in the National Center for Biotechnology Information (see text footnote 1) for the 18S rRNA gene and ITS1–5.8S rDNA–ITS2 region sequence.

Etymology: The species epithet ‘tropica’ refers to the climate region in which the strain was isolated.

Remarks: *C. tropica* is similar in shape and cell size to the most closely phylogenetically related *C. onubensis*, *C. actinabiotis* and some other species: *C. cimbrica*, *C. greatwallensis* (Supplementary material 1). Exact identification is possible only by using 18S rDNA and ITS2 sequence analysis.

Distribution: Known from the type locality and the bark of *Syzygium nervosum* in Indonesia (Java) for the strain KN-2011-T3.

**FIGURE 8:**
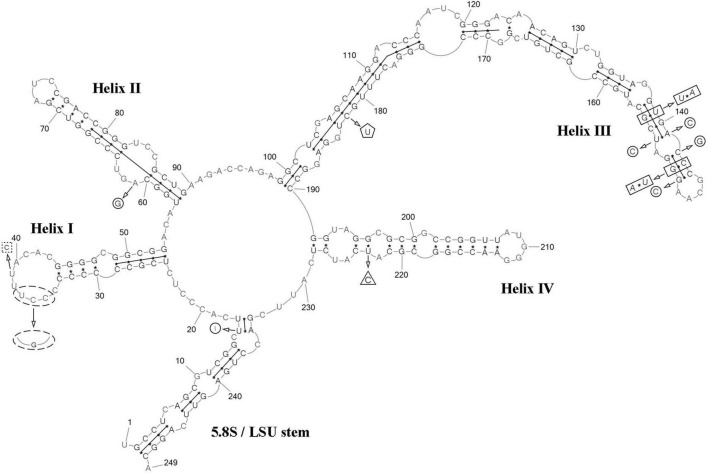
Predicted secondary structure of the ITS2 sequence of the *Coccomyxa tropica*, strain VP521. Base numbering is indicated every 10 bases. The four helices are numbered with Roman numerals. The barcode region of the ITS2 sensu Darienko et al. (2015) is shown by lines inside the secondary structure. Deletions and single bases for *Coccomyxa* sp. KN-2011-T3 are shown by a square with dotted sides. hCBC outside the barcode region for “*C*. *onubensis*” ACCV1 is shown by a triangle. CBCs within the barcode region for “*C*. *actinabiotis*” CCAP 216/25 are shown by a rectangle. hCBC within the barcode region for “*C*. *actinabiotis*” CCAP 216/25 is shown by a pentagon. Deletions and single bases within the barcode region for “*C*. *actinabiotis*” CCAP 216/25 are shown by a circle. Deletions and single bases outside the barcode region for “*C*. *actinabiotis*” CCAP 216/25 and “*C*. *onubensis*” ACCV1 are not shown.

ITS2 analysis: We analyzed the ITS2 secondary structure of *C*. *tropica* and strains from the clade *C*. *polymorpha*: *Coccomyxa* sp. KN-2011-T3, “*C*. *onubensis*” ACCV1 and “*C*. *actinabiotis*” CCAP 216/25 (Figures 2, 8). The VP521 and KN-2011-T3 differed only by deletions and single bases outside the barcode region. One hCBC (G–U → G–C) in the helix IV (outside the barcode region) differed *C*. *tropica* from “*C*. *onubensis*” ACCV1. Comparison of the secondary structure of ITS2 for *C*. *tropica* and “*C*. *actinabiotis*” CCAP 216/25 showed several distinctive features within the barcode region: two CBCs (U–G → A–U; C–G → U–A) and one hCBC (G–C → G–U) in the helix III; total six single bases in the 5.5S/LSU stem, helices II and III. These differences in ITS2 between the strain VP521 and the CCAP 216/25 can be considered interspecific variations.

#### 3.1.4 *Coccomyxa cattiensis* Maltsev et Kezlya

**FIGURE 9:**
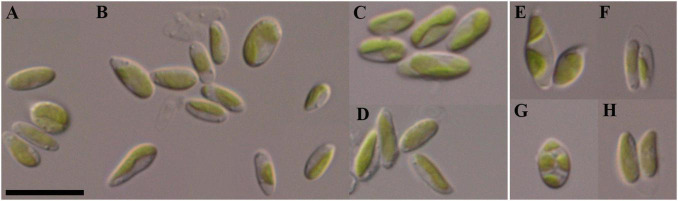
Nomarski interference micrographs of *Coccomyxa cattiensis*, strain VP451 in culture. Scale bar = 10 μM. **(A–D)** Variable shapes of culture cells, age 2 weeks; **(E–H)** asexual reproduction through cell division with the formation of 2–4 autospores, age 4 weeks.

Diagnosis (Figure 9): Mature vegetative cells solitary, ovoid, ellipsoidal to long ellipsoidal, sometimes slightly curved, asymmetrical, pointed on one side, without mucilaginous sheath, cell size 3.6–5.7 (7.1) μm in length 1.5–2.6 μm in width. Chloroplast parietal, trough-shaped, thin, straight or with small concavity in the middle, covering half or 2/3 of the cell volume. Pyrenoid is absent. Cell wall is thin. Reproduction by 2–4 autospores. Protoplast division is transverse or oblique. Liberation of autospores through the rupture of mother cell wall at one end. The released autospores have the same morphology as mature vegetative cells.

Holotype (designated here): Material from the authentic strain VP451 is stored in the IPPAS collection of microalgae and cyanobacteria (WDCM 596), Moscow, Russian Federation (metabolically inactive cryopreserved culture). Preserved specimen with unfixed, dried cells (in a metabolically inactive state) from a large drop of a batch of strain VP451 added to watercolor paper (Figure 9), deposited at the MHA (Herbarium, Main Botanical Garden, Botanicheskaya Str. 4, Moscow, 127276, Russia) under the designation *Coccomyxa cattiensis* Vietnam Kezlya strain VP451. The culture was also deposited and maintained as an active culture under the designation VP451 in the Culture and Barcode Collection of Microalgae and Cyanobacteria “Algabank” (WDCM 1318) at K.A. Timiryazev Institute of Plant Physiology RAS. Also, the strain is conserved as a formaldehyde-fixed sample.

Reference strain: VP451, paratype strain: VP449.

Type locality: Forest soil, Cát Tiên National Park, Đồng Nai Province, Vietnam (11°23.917′ N, 107°22.523′ E): soil sample KT55 collected on 07 June 2019.

Sequence data: Accession numbers PV748125 for the strain VP451, and PV748124 for the strain VP449 in the National Center for Biotechnology Information (see text footnote 1) for the 18S rRNA gene and ITS1–5.8S rDNA–ITS2 region sequences.

Etymology: The species epithet “cattiensis” refers to the name of Cát Tiên National Park, where this species was observed.

Remarks: *C. cattiensis* differs in size and shape of cells from a closely related in the phylogeny *C. antarctica*. The cells of the latter are larger: 8.0–12.0 μm in length, 4.0–7.0 in μm width, ovoid to ellipsoidal in the form vs. 3.6–7.1 μm in length, 1.5–2.6 μm in width in *C. cattiensis* ovoid to long ellipsoidal in the form. For sister taxon in a phylogenetic lineage, *C*. *parasitica*. South showed a very variable in shape from cells: spherical to elliptical, oblong or sickle-shaped, frequently with an attenuated hyaline tip, whereas spherical and sickle-shaped not observed in new species. Several species are very similar in morphology: *C. vinatzeri* and *C. fottii* (Supplementary material 1). There are no clear differences in the size and shape of cells or the structure of the chloroplast in these species. Exact identification is possible only by using 18S rDNA and ITS2 sequence analysis.

Distribution: As yet known only from the type locality. Strain VP449 isolated from forest soil, sample KT30, Cát Tiên National Park, Đồng Nai Province, Vietnam (11°25.725′ N, 107°25.650′ E) (Table 1).

**FIGURE 10:**
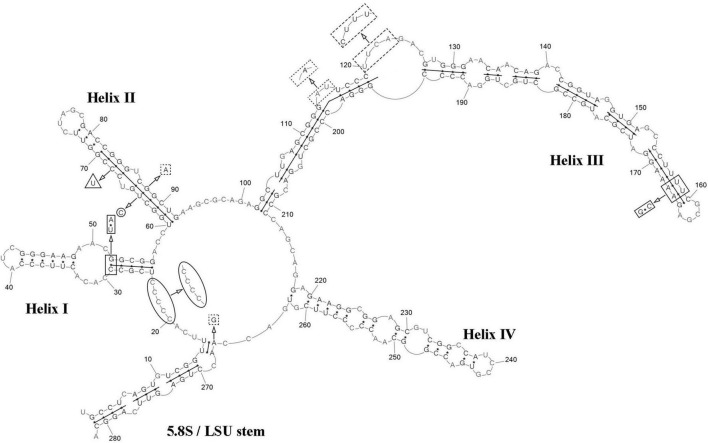
Predicted secondary structure of the ITS2 sequence of the *Coccomyxa cattiensis*, strain VP451. Base numbering is indicated every 10 bases. The four helices are numbered with Roman numerals. Differences with the strain VP449 are shown by circle and ellipse. The barcode region of the ITS2 sensu Darienko et al. (2015) is shown by lines inside the secondary structure. Differences in characteristics within the barcode region for *Coccomyxa antarctica* Ua6 (FACHB-2140) are shown by nucleotides outside the secondary structure. CBCs are shown by rectangles, and hCBC is shown by triangles. Deletion and single bases are shown by squares with dotted sides. Deletions and single bases outside the barcode region are not shown.

ITS2 analysis: The present study detected several distinctive features between *C*. *cattiensis vs C*. *antarctica* Ua6 (FACHB-2140) inside the barcode region: one CBC in the helix I (C–G → U–A) and one CBC in the helix III (U–A → C–G), one hCBC (C–G → U–G) in the helix II, and several deletions and single bases in the 5.5S/LSU stem, helices II and III (Figure 10). Thus, the described species *C*. *cattiensis* has high evolutionary isolation from *C*. *antarctica*. Strains VP451 and VP449 differed by one deletion outside the barcode region and one U→C transition in helix II.

### 3.2 Growth parameters

**FIGURE 11:**
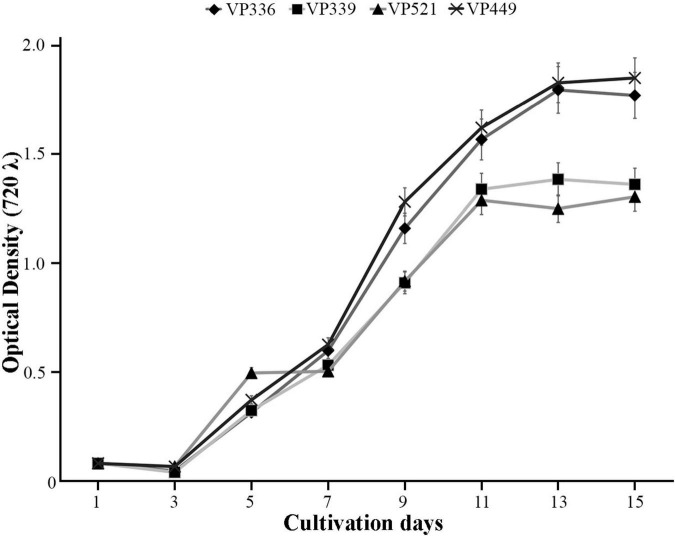
Growth kinetics of *Coccomyxa* strains displaying changes in an optical density (OD), arithmetic means ± standard errors, *n* = 3.

The novel *Coccomyxa* strains were used in cultivation experiments to evaluate growth dynamics. The initial cultures had OD_720_ of 0.08. The growth curves showed typical sigmoid character for all cultures (Figure 11). The lag phase was observed during the initial 3 days of cultivation for all strains. After day 3, all cultures entered the exponential phase with a steady increase in optical density till day 11 for VP339 and VP521 strains or day 13 for VP336 and VP449 strains. The cultures subsequently slowed their growth and entered a stationary phase, during which OD_720_ and cell counts remained almost constant. Starting from day 7, OD_720_ was significantly lower for VP339 and VP521 than VP336 and VP449 (Figure 11). The observed character of biomass accumulation by *Coccomyxa* strains suggests that the strain *C*. *cattiensis* VP449 had a higher optical density of culture.

### 3.3 Fatty acid profiles

Analysis of the biomass at the stationary phase revealed the domination of PUFAs for all strains, notably 18:2n-6 linoleic acid (within the range of 4.54–21.69% of total FAs) and 18:3n-3 α-linolenic (26.78–47.02%) (Tables 2, 3; Supplementary material 2). The highest content of PUFAs (75.11% of the total FA content corresponding to the total yield of 655.55 mg L^–1^) was observed for *C*. *fusiformis* VP339. In addition, *C*. *subellipsoidea* VP336, *C*. *tropica* VP521, and *C*. *cattiensis* VP449 accumulated high amounts of saturated 16:0 palmitic (22.01–31.16%) and monounsaturated 16:3n-3 roughanic (7.70–16.12%) acids. Small amounts of monounsaturated 16:1n-9 hypogeic (≤ 0.65%) and 16:1n-7 palmitoleic (≤ 3.18%) acids were determined in all strains, whereas *C*. *subellipsoidea* VP336, *C*. *tropica* VP521 and *C*. *cattiensis* VP449 also produced small amounts of the long-chain 20:4n-6 arachidonic acid (≤ 1.90%). We determined the long-chain omega-3 20:5n-3 eicosapentaenoic acid in small concentrations (0.85%) exclusively in *C*. *subellipsoidea* VP336 biomass. In general, novel strains were marked by a high content of omega-3 FAs. The greatest concentration of omega-3 acids (62.64%), including 18:3n-3 α-linolenic acid, was observed in the strain *C*. *subellipsoidea* VP336. At the same time, the VP339 culture contained the highest total yield of omega-3 acids, 485.28 mg L^–1^ (Table 3). New species also had specific features in FA profiles. *C*. *fusiformis* VP339 differed from the other species by the highest concentrations of 16:2n-6 hexadecadienoic (*F*_3_,_8_ = 15.83, *P* = 0.001), 16:4n-3 hexadecatetraenoic (*F*_1_,_4_ = 74.14, *P* = 0.001) and 18:1n-9 oleic (*F*_3_,_8_ = 15.83, *P* = 0.001) acids and low content of 16:0 palmitic (*F*_3_,_8_ = 15.83, *P* = 0.001) and 16:3n-3 roughanic acids (*F*_3_,_8_ = 15.83, *P* = 0.001) (Table 2). *C*. *cattiensis* VP449 had the highest content of 16:0 palmitic, 18:2n-6 linoleic and omega-6 FAs (*F*_3_,_8_ = 15.83, *P* = 0.001 for all values). *C*. *subellipsoidea* VP336 was characterized by maximum value of omega-3 FAs (*F*_3_,_8_ = 6.57, *P* = 0.015 with VP339; *F*_3_,_8_ = 5.79, *P* = 0.021 with VP521; *F*_3_,_8_ = 15.83, *P* = 0.001 with VP449) and the highest ratio of omega-3 and omega-6 FAs (9.4:1 in the total yield).

**TABLE 2:** Fatty acid composition of new *Coccomyxa* strains.

Fatty acid	*C*. *subellipsoidea* VP336	*C*. *fusiformis* VP339	*C*. *tropica* VP521	*C*. *cattiensis* VP449
14:0 Myristic	1.22^ABC^ ± 0.07	0.16^A^ ± 0.01	0.16^B^ ± 0.02	0.24^C^ ± 0.01
16:0 Palmitic	22.53^A^ ± 0.37	13.59^AB^ ± 0.31	22.01^B^ ± 0.59	31.16^AB^ ± 1.03
16:1n-9 Hypogeic	0.16^Aa^ ± 0.01	0.29^aB^ ± 0.02	0.65^AB^ ± 0.04	0.11^B^ ± 0.02
16:1n-7 Palmitoleic	3.18^AB^ ± 0.09	2.49^Aa^ ± 0.09	2.10^aB^ ± 0.09	1.29^AB^ ± 0.04
16:2n-6 7,10-Hexadecadienoic	0.26^A^ ± 0.02	4.95^A^ ± 0.14	2.90^A^ ± 0.09	3.83^A^ ± 0.11
16:3n-3 Roughanic	10.77^A^ ± 0.33	1.44^A^ ± 0.06	16.12^A^ ± 0.39	7.70^A^ ± 0.21
16:3n-6 4,7,10-Hexadecatrienoic		4.96 ± 0.14		
16:4n-3 4,7,10,13-Hexadecatetraenoic	3.95^A^ ± 0.14	17.16^A^ ± 0.57		
18:0 Stearic	1.74^A^ ± 0.07	0.31^A^ ± 0.03	1.04^A^ ± 0.04	2.91^A^ ± 0.10
18:1n-9 Oleic	1.73^A^ ± 0.05	8.02^A^ ± 0.24	5.60^A^ ± 0.14	3.03^A^ ± 0.07
18:1n-7 Vaccenic			0.48^a^ ± 0.04	0.68^a^ ± 0.04
18:2n-6 Linoleic	4.54^AB^ ± 0.14	9.48^A^ ± 0.12	8.88^B^ ± 0.24	21.69^AB^ ± 0.34
18:3n-6 γ-Linolenic		0.11^Aa^ ± 0.02	0.05^aB^ ± 0.01	0.41^AB^ ± 0.02
18:3n-3 α-Linolenic	47.02^A^ ± 0.76	32.83^A^ ± 0.64	39.92^A^ ± 1.14	26.78^A^ ± 0.62
18:4n-3 Stearidonic	0.05^A^ ± 0.01	4.18^A^ ± 0.12		
20:0 Arachidic	0.10^A^ ± 0.01	0.03^A^ ± 0.01		
20:4n-6 Arachidonic	1.90^AB^ ± 0.06		0.09^A^ ± 0.01	0.17^B^ ± 0.02
20:5n-3 Eicosapentaenoic	0.85 ± 0.20			
SFA	25.59^A^ ± 0.36	14.09^AB^ ± 0.29	23.21^B^ ± 0.60	34.31^AB^ ± 1.0
MUFA	5.07^A^ ± 0.08	10.80^AB^ ± 0.23	8.83^AB^ ± 0.17	5.11^B^ ± 0.09
PUFA	69.34^A^ ± 1.35	75.11^aB^ ± 1.39	67.96^ab^ ± 1.75	60.58^ABb^ ± 0.81
Omega-3	62.64^Aab^ ± 1.23	55.61^aB^ ± 1.26	56.04^bC^ ± 1.53	34.48^ABC^ ± 0.74
Omega-6	6.70^A^ ± 0.12	19.50^A^ ± 0.13	11.92^A^ ± 0.23	26.10^A^ ± 0.29
Omega-3/omega-6	9.3	2.6	4.7	1.3

The data are reported as the mean (% of total fatty acids) ± standard error from three independent biological replicates.

^A,B,C^Mean with unlike superscript in row differ significantly (*P* ≤ 0.01). ^a,b^Mean with unlike superscript in row differ significantly (*P* ≤ 0.05).

**TABLE 3:** Fatty acid composition of new *Coccomyxa* strains.

Fatty acid	*C*. *subellipsoidea* VP336	*C*. *fusiformis* VP339	*C*. *tropica* VP521	*C*. *cattiensis* VP449
14:0 Myristic	4.65^AB^ ± 0.16	2.78^AB^ ± 0.11	0.97^B^ ± 0.03	0.57^A^ ± 0.01
16:0 Palmitic	86.0^A^ ± 3.06	237.0^AB^ ± 6.66	131.0^AB^ ± 4.51	74.57^B^ ± 1.78
16:1n-9 Hypogeic	0.61^Aa^ ± 0.02	5.11^AB^ ± 0.13	3.89^AB^ ± 0.05	0.27^aB^ ± 0.01
16:1n-7 Palmitoleic	12.14^A^ ± 0.27	43.51^AB^ ± 0.71	12.50^B^ ± 0.28	3.08^AB^ ± 0.03
16:2n-6 7,10-Hexadecadienoic	1.01^A^ ± 0.04	86.61^A^ ± 1.50	17.21^A^ ± 0.98	9.19^A^ ± 0.53
16:3n-3 Roughanic	41.10^A^ ± 0.96	25.26^A^ ± 0.31	95.96^A^ ± 1.9	18.44^A^ ± 0.44
16:3n-6 4,7,10-Hexadecatrienoic		86.61 ± 0.94		
16:4n-3 4,7,10,13-Hexadecatetraenoic	15.06^A^ ± 0.53	299.49^A^ ± 9.77		
18:0 Stearic	6.67^A^ ± 0.20	5.34^AB^ ± 0.09	6.19^B^ ± 0.08	6.96^B^ ± 0.05
18:1n-9 Oleic	6.61^A^ ± 0.11	139.87^AB^ ± 2.18	33.30^AB^ ± 0.83	7.25^B^ ± 0.15
18:1n-7 Vaccenic			2.88^A^ ± 0.03	1.63^A^ ± 0.05
18:2n-6 Linoleic	17.32^AB^ ± 0.33	165.40^AB^ ± 3.99	52.87^A^ ± 0.72	51.90^B^ ± 0.64
18:3n-6 γ-Linolenic		1.89^A^ ± 0.03	0.30^A^ ± 0.01	0.98^A^ ± 0.01
18:3n-3 α-Linolenic	179.44^A^ ± 3.51	572.85^A^ ± 11.66	237.65^A^ ± 5.17	64.09^A^ ± 0.84
18:4n-3 Stearidonic	0.19^A^ ± 0.01	72.94^A^ ± 0.65		
20:0 Arachidic	0.37^A^ ± 0.01	0.56^A^ ± 0.02		
20:4n-6 Arachidonic	7.24^AB^ ± 0.36		0.55^A^ ± 0.01	0.42^B^ ± 0.01
20:5n-3 Eicosapentaenoic	3.24 ± 0.05			
SFA	97.69^A^ ± 3.42	245.68^AB^ ± 6.86	138.16^AB^ ± 4.60	82.10^B^ ± 1.84
MUFA	19.36^A^ ± 0.40	188.49^AB^ ± 2.96	52.57^AB^ ± 1.18	12.23^B^ ± 0.24
PUFA	264.60^A^ ± 5.72	1311.05^A^ ± 28.72	404.54^A^ ± 8.73	145.02^A^ ± 2.42
Omega-3	239.03^A^ ± 5.01	970.54^A^ ± 22.37	333.61^A^ ± 7.03	82.53^A^ ± 1.27
Omega-6	25.57^AB^ ± 0.72	340.51^AB^ ± 6.36	70.93^A^ ± 1.71	62.49^B^ ± 1.19
Omega-3/omega-6	9.4	2.9	4.7	1.3

The data are reported as the mean (mg L^–1^) ± standard error from three independent biological replicates.

^A,B^Mean with unlike superscript in row differ significantly (*P* ≤ 0.01). *^a^*Mean with unlike superscript in row differ significantly (*P* ≤ 0.05).

## 4 Discussion

The results of recent studies of *Coccomyxa* using molecular methods have shown that identification based solely on morphological characteristics is possible up to the genus level (Darienko et al., 2015; Malavasi et al., 2016; Barcytë and Nedbalová, 2017; Cao et al., 2018b). Species identification is possible only with an integrative approach (Darienko et al., 2015). The experiments of Darienko et al. (2015) demonstrated changes in morphological characteristics under different physiological conditions. *Coccomyxa* cells changed size and shape depending on the concentration of NaCl in the medium. Morphological plasticity has also been shown for *C*. *silvae-gabretae* from Plešné Lake in the Czech Republic (Barcytë and Nedbalová, 2017). Under natural conditions, the species had elongated spindle-shaped cells, while under laboratory conditions, these features disappeared, and the cells acquired an ellipsoidal shape. Therefore, morphometric characteristics cannot be considered diagnostic features for *Coccomyxa* species identification.

The formation of a one-side mucilage cap was previously considered a distinctive feature of the genus (Andreyeva, 1998). Later, as a result of the phylogenetic analysis of Darienko et al. (2015), it was shown that two closely related genera, *Coccomyxa* and *Pseudococcomyxa*, represent a monophyletic group. *Coccomyxa* and *Pseudococcomyxa* species were previously separated according to the ability of cells to form mucilage (Andreyeva, 1998). In this regard, the ability of mucilage formation in *Coccomyxa*-like strains under starvation conditions was tested (Darienko et al., 2015). The authors investigated nine strains representing all the phylogenetic lineages of *Coccomyxa* and showed that after 6 weeks of starvation, only 2 out of 9 strains were observed to produce mucilaginous sheath. Thus, it has been proven that mucilage formation is not characteristic of all *Coccomyxa* species. In recent years, the absence of mucilage has been shown for several new species, such as *C*. *antarctica* (Cao et al., 2018a), *C*. *greatwallensis* (Cao et al., 2018b), *C*. *fottii*, *C*. *silvae-gabretae* (Barcytë and Nedbalová, 2017).

Determining species boundaries is based on DNA sequence analysis, considering morphology and ecology (integrative approach). The first paper on a detailed study of *Coccomyxa* was published by Darienko et al. (2015). Based on the analysis of the molecular structure of 18S rRNA and ITS2 markers of 41 strains from public and working collections, it was proposed to distinguish 7 *Coccomyxa* species. Later, as additional criteria for the species, Malavasi et al. (2016) proposed to evaluate environmental data. As a result of the analysis of 61 sequences (18S rDNA and ITS1–5.8S rDNA–ITS2) of *Coccomyxa*, it was shown that the living stage and habitat ecological characteristics of species are consistent with the phylogenetic lineages (Malavasi et al., 2016). Other authors also support this approach (Barcytë and Nedbalová, 2017; Sciuto et al., 2019).

All studied strains were isolated from the upper 5 cm layer of soil on four different sampling sites, of which 3 were forests and 1 was a dry swamp (Figure 1 and Table 1). Only *C*. *cattiensis* was found at two points; the remaining species were recorded once. The described species have a typical morphology for *Coccomyxa*: small cells with ellipsoidal or ovoid shape, parietal chloroplast shape without a pyrenoid, and oblique cell division. A specific characteristic of *C*. *fusiformis* is the presence of cells with large sizes and spindle-like shape, reminiscent of the crescent-shaped cells of *Monoraphidium* (Figure 5). We investigated all strains under starvation conditions within 10 weeks with cultivation in a 1:20 diluted 3N-BBM + V medium containing 1% glucose. The mucilage production in the culture cells was not observed.

Our phylogenetic analysis includes 18S rDNA and ITS1–5.8S rDNA–ITS2 sequences of 80 *Coccomyxa* strains. The tree topology is consistent with previous studies (Darienko et al., 2015; Malavasi et al., 2016; Barcytë and Nedbalová, 2017; Sciuto et al., 2019). *Coccomyxa* species form a monophyletic group, and previously designated clades are supported (Figure 2). New species described in this study form sister lineages to clades G and *C. subellipsoidea* (*C. fusiformis*), clade I (*C. tropica*) sensu Malavasi et al. (2016) and clade “*C. parasitica*” (*C. cattiensis*) sensu Sciuto et al. (2019) (Figure 2).

**FIGURE 12:**
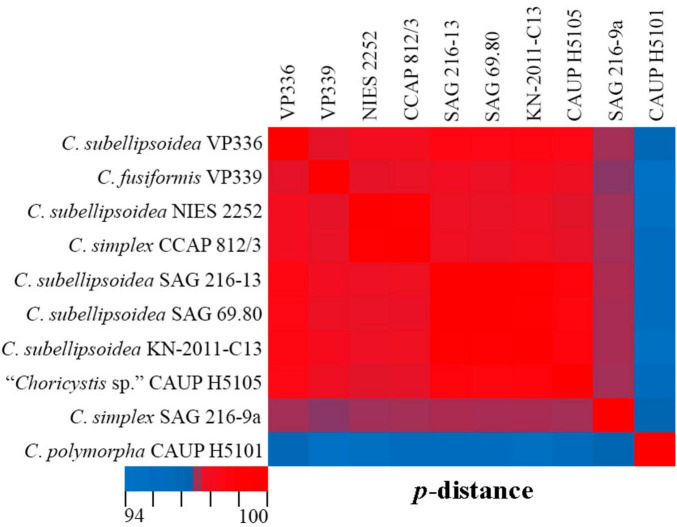
Pairwise nucleotide identity (*p*-distance) heatmap based on the 18S rDNA–ITS1–5.8S rDNA region (2,203 bp) showing 10 *Coccomyxa* strains including VP336 and VP339. Red indicates a high nucleotide identity, and blue indicates a lower nucleotide identity value. The scale bar represents color codes of nucleotide identity values, showing sequence identity percentage. See Supplementary material 3, Table 1 for the exact *p*-distance values.

Phylogenetic positions in the tree are consistent with the ecological differentiation of *Coccomyxa* lineages, as suggested by Malavasi et al. (2016). Most strains in the clade *C. subellipsoidea* are photobionts of soil lichens, rarely epiphytic. Terrestrial and epilithic taxa represent neighboring clades E, G, and H (Malavasi et al., 2016). New strain *C. subellipsoidea* VP336 forms the lineage next to the terrestrial taxa of the clade E: *Coccomyxa* sp. NIES 2252 (unknown habitat), *Coccomyxa* sp. IB-GF-12 (soil habitat). New species *C. fusiformis* forms the lineage next to the epilithic strain CAUP H5105 from the clade G in Malavasi et al. (2016). The evolutionary distance matrix based on 18S rDNA–ITS1–5.8S rDNA region showed that *C. subellipsoidea* shared 99.0–99.3% similarities with other *Coccomyxa* strains from the clade *C. subellipsoidea* sensu Darienko et al. (2015) (Figure 12) and 94.9–98.7% similarities with *Coccomyxa* species from other clades. The *p*-distance matrix showed that *C. fusiformis* shared 98.8–99.1% similarities with strains from the clade *C. subellipsoidea*, and 94.5–98.9% similarities with strains from clades G, *C*. *simplex* and *C*. *polymorpha* (Figure 12).

**FIGURE 13:**
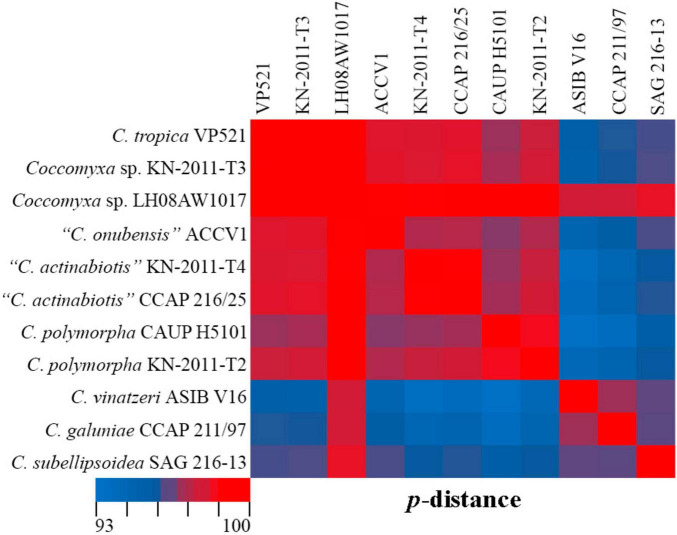
Pairwise nucleotide identity (*p*-distance) heatmap based on the 18S rDNA–ITS1–5.8S rDNA region (2,227 bp) showing 11 *Coccomyxa* strains including VP521. Red indicates a high nucleotide identity, and blue indicates a lower nucleotide identity value. The scale bar represents color codes of nucleotide identity values, showing sequence identity percentage. See Supplementary material 3, Table 2 for the exact *p*-distance values.

*C. tropica* complements clade I with high statistical support (92ML/0.94BI), most closely related to the tropical epiphytic strain *Coccomyxa* sp. KN-2011-T3. Pairwise comparisons of 18S rDNA–ITS1–5.8S rDNA region with taxa from the clade *C*. *polymorpha* sensu Darienko et al. (2015) showed that *C. tropica* was 98.0–100% similar *Coccomyxa* strains (Figure 13). The highest sequence similarity (100%) was recorded with strain *Coccomyxa* sp. KN-2011-T3. Additionally, the absence of CBC, hCBC, and single bases within the barcode region of the ITS2 suggests that strain KN-2011-T3 belongs to *C. tropica* as *C.* cf. *tropica*. Another strain in this clade, *Coccomyxa* sp. LH08AW1017 was isolated from soil in Germany. Thus, the clade I was formed by *Coccomyxa* from terrestrial habitat.

**FIGURE 14:**
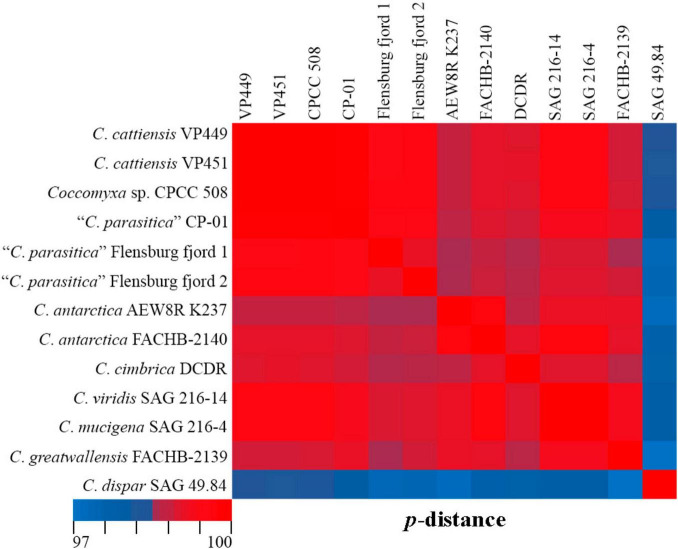
Pairwise nucleotide identity (*p*-distance) heatmap based on the 18S rRNA gene (1,788 bp) showing 13 *Coccomyxa* strains including VP449 and V451. Red indicates a high nucleotide identity, and blue indicates a lower nucleotide identity value. The scale bar represents color codes of nucleotide identity values, showing sequence identity percentage. See Supplementary material 3, Table 3 for the exact *p*-distance values.

*C. cattiensis* is represented by VP449 and VP451, which are closely related to the free-living acid-tolerant strain *Coccomyxa* sp. CPCC 508, isolated from the metal-contaminated area in Boomerang Lake, near Red Lake (ON, Canada) (Verma et al., 2009). The highest 18S rRNA sequence similarity (100%) between *C. cattiensis* and *Coccomyxa* sp. CPCC 508 (Figure 14) suggests this strain belongs to the described species as *C.* cf. *cattiensis*. Neighboring sequences from the clade *C*. *parasitica* sensu Sciuto et al. (2019) belong to strains infesting in mussels and probably represent separate species from *C. cattiensis*. Strain CP-01 was isolated from horse mussel *Modiolus kurilensis* (Sokolnikova et al., 2016). The two Flensburg fjord strains (1 and 2) were also attributed to the species *C. parasitica* and characterized as a facultative parasite blue mussel *Mytilus edulis* (Rodríguez et al., 2008). *C. cattiensis* showed sequence similarity of 99.6–99.7% to “*C*. *parasitica*” Flensburg fjord (1 and 2) and 99.4% to *C*. *antarctica* FACHB-2140 (Figure 14). Given the ecological and phylogenetic characteristics, the new species *C. cattiensis* is separated from other members of the clade *C*. *parasitica* sensu (Sciuto et al., 2019).

Obtained FA profiles were compared with the published evidence on FA profiles of other *Coccomyxa* strains in Supplementary material 2. The analysis compared the FA profiles of *Coccomyxa* strains representing different habitats and varying culture times. The study included strains *C*. *elongata* SAG 216-3a, *C*. *mucigena* SAG 216-4, *C*. s*implex* SAG 216-8, SAG 216-9a, *C*. *solorinae* SAG 216-5, and *C*. *subellipsoidea* SAG 216-7, which maintained in culture since 1954, *C*. *subellipsoidea* SAG 69.80 since 1971, *C*. *dispar* SAG 49.84 since 1983 and *C*. *elongata* MZ–Ch64 since 2015. *Coccomyxa* strains of lichen photobionts from the lineage *C*. *solorinae* revealed a high content of PUFAs in the range of 55.60–75.30% of total fatty acids and a low concentration of MUFAs (Lang et al., 2011). Free living strains from different habitats and clades (freshwater *C*. *elongata* MZ–Ch64, epiphyte *C*. *avernensis* SAG 216-1 and *Coccomyxa* sp. SAG 2040) showed enrichment with MUFAs: 60.09% (Maltsev et al., 2019), 46.02 and 42.94% (Lang et al., 2011), respectively. The leader in SFA accumulation was the strain *C*. *melkonianii* SCCA048 (45.50%) from a river highly polluted by heavy metals (Soru et al., 2019). Conversely, this FA group’s minimum values (8.83–9.64%) are shown for *Coccomyxa* strains of lichen photobionts from the lineage *C*. *subellipsoidea* (Lang et al., 2011). Within PUFAs special attention is paid to omega-3 and omega-6 FAs since they are characterized by different features and play an essential role in providing regular activity for humans and animals (Horrocks and Yeo, 1999). New species from the tropical forest soil (*C*. *tropica*) together with strains *C*. *subellipsoidea* SAG 216-7 and SAG 69.80 accumulated omega-3 FAs in the range of 54.75–62.64% with maximum value for strain *C*. *subellipsoidea* VP336. Compared to oils obtained from other productive green algae strains (Maltsev and Maltseva, 2021), oil produced by the strain VP336 has a similar content of omega-3 FAs with *Chlamydomonas reinhardtii* strains SAG 11-32a, SAG 11-32b, and SAG 73.72 (62.80–63.81% of omega-3). On the other hand, new *Coccomyxa* strains are characterized by a more minor concentration of omega-3 FAs in contrast to *Chlamydomonas hydra* strain SAG 11-6b and SAG 11-6c (76.63–85.26%, respectively). *Coccomyxa* strains (SAG 216-14, SAG 2040) from the clade *C*. *viridis* revealed a high content of omega-6 FAs (40.0%, 36.54%) and the lowest content of omega-3 FAs (Supplementary material 2). To sum it up, new *Coccomyxa* strains accumulate remarkably high amounts of PUFAs (up to 655.55 mg L^–1^) compared to *Coccomyxa onubensis* with 161.2 mg L^–1^ (Bermejo et al., 2018) and such green algae strain as *Coelastrella multistriata* MZ–Ch23 with 300.1 mg L^–1^ (Maltsev et al., 2021). The ratio of omega-3 and omega-6 FAs is essential for the biotechnological use of algal biomass. Most notably, it should have high omega-3 FA concentrations accompanied by low omega-6 FA content. Different values of this ratio characterize *Coccomyxa* strains: extra low content omega-3 over omega-6 with the factor of 0.148–0.969 (strains from the clade *C*. *viridis*), slight predominance omega-3 over omega-6 by 1.551–3.1-fold (strains from the clade *C*. *simplex*, except MZ–Ch64, SAG 216-2, SCCA048), significant predominance omega-3 over omega-6 by 2.637–9.342-fold (*C*. *fusiformis*, *C*. *tropica* and strains from the clade *C*. *subellipsoidea*, except NIES 2166 from Antarctica). We found the maximum value of the omega-3/omega-6 ratio in strain *C*. *subellipsoidea* VP336 (Table 2; Supplementary material 2). Generally, the analysis shows equitable trends in fatty acid composition among strains from similar habitats and close phylogenetic clades. At the same time, the long-term culture time of *Coccomyxa* is not accompanied by a loss of specific biochemical characteristics. For example, strains *C*. *subellipsoidea* SAG 216-7 and SAG 69.80 are lichen photobionts and belong to the same phylogenetic subclade (Figure 2), but were isolated from geographically distant areas (Finland and Germany) and maintained as cultures for different times (since 1954 and 1970). However, these strains are characterized by similar values of omega-3 FA content (56.39% and 54.75%) and omega-3/omega-6 ratio (5.3 and 4.4). Among other trebouxiophycean species, the highest omega-3 to omega-6 ratio was observed in *Neglectella solitaria* SAG 83.80 (30.6:1), *Oocystis parva* SAG 82.80 (28.7:1) and *Koliella longiseta* SAG 470-1 (9.1:1). The opposite excess of omega-6 over omega-3 fatty acids has been reported to *Parachlorella kessleri* SAG 10.80 (14.4:1), *Micractinium pusillum* SAG 13.81 (10.3:1) and *Myrmecia bisecta* SAG 2043 (9.3:1) (Lang et al., 2011; Maltsev and Maltseva, 2021).

A well-balanced proportion of omega-3 and omega-6 FAs and a high content of 18:3n-3 α-linolenic acid determine the possibility of using biomass of new *Coccomyxa* species from forest soils of Vietnam (especially strains VP336 and VP521) as a component of highly effective food additives or forage in aquaculture and farming. Enrichment of feed with fatty acids is a new strategy for agriculture (Ma et al., 2019). The high content of SFAs and PUFAs (up to 94.89%) positions the novel strain *C*. *cattiensis* VP449 as a promising feed additive that promotes methane mitigation from ruminants (Beauchemin et al., 2022). The favorable effect of the algal biomass inclusion in the diet was observed for growing various birds and farm animals (Lamminen et al., 2019; Petrolli et al., 2019). Fortification of the cows’ diet with microalgae-based feed resulted in the successful enrichment of milk with omega-3 docosahexaenoic acid up to 4.5–6.4 mg/100 mL milk (Moran et al., 2019). In the future, it will also be necessary to carry out cultivation experiments to evaluate growth dynamics and the impact of stress factors on fatty acid profiles and lipid productivity.

Thus, phylogenetic analysis and considering habitat ecological characteristics confirm the description of three new species of *Coccomyxa*. The recognition and description of these novel species suggest that the algal flora of Vietnam’s soil habitats has yet to be fully documented.

## 5 Conclusion

Investigations of soil green algae using molecular methods are still minimal. Using an integrative approach, we have described three new *Coccomyxa* species from an understudied region. Phylogenetic analysis based on the 18S rRNA gene indicates that new species were from separate lineages. The fatty acid composition of the studied soil *Coccomyxa* strains showed an increased content of palmitic, α-linolenic, and linoleic acids. Given the fatty acid content of the strain’s biomass, they are a promising natural source for feed production, promoting methane mitigation from ruminants and the nutraceutical and pharmaceutical industries. In future studies, adjustments to lighting intensity, the composition of the medium, and two-stage cultivation will be examined to optimize cultivation conditions and improve the productivity of *Coccomyxa* strains.

## Data Availability

The datasets presented in this study can be found in online repositories. The names of the repository/repositories and accession number(s) can be found in the article/Supplementary material.

## Footnotes

1
https://www.ncbi.nlm.nih.gov
