# Supplementary material for: Phylogeny and fatty acid profiles of new Coccomyxa (Chlorophyta) species from soils of Vietnam
Source: Front Microbiol. 2025 Jul 14;16:1517865. doi: 10.3389/fmicb.2025.1517865 (PMC12301341; doi:10.3389/fmicb.2025.1517865)
Supplement: Supplementary file 1 [file Table_1.docx]

SUPPLEMENTARY MATERIAL 1 The main morphological features of *Coccomyxa* species studied in this research and similar taxa.

| Species | Mature cells form | Cells size (length (μm) × width (μm) | Chloroplast | Reproduction | Habitat | Reference |
| --- | --- | --- | --- | --- | --- | --- |
| *Coccomyxa cattiensis* Maltsev et Kezlya  (strains VP451, VP449) | Solitary, ovoid to long ellipsoidal, sometimes slightly curved, asymmetrical, pointed  on one side, without mucilaginous sheath | 3.6–7.1 × 1.5–2.6 | Parietal, trough-shaped, thin, straight or with small concavity in the middle, covering half or 2/3 of the cell volume | 2–4 autospores | Soil, tropic forest, Vietnam | This study |
| *Coccomyxa tropica* Maltsev et Kezlya  (strain VP521) | Solitary, ellipsoidal, sometimes ovoid, without mucilaginous sheath | 3.3–6.9 × 1.5–2.6 | Parietal, trough-shaped, thick, covering about half of the cell volume. Lipid droplets present in the cytosol | 2–4 autospores | Soil, tropic forest, Vietnam | This study |
| *Coccomyxa fusiformis* Maltsev et Kezlya  (strain VP339) | Solitary, elongated-oval, very often pointed on both sides, spindle-shaped, slightly curved, asymmetric, without mucilaginous sheath, | 8.0–16.2 × 1.9–3.4 | Parietal, thin, trough-shaped or ribbon-like, with waved margin in large cells, covering about half of the cell volume | 2–4 autospores | Soil, dry swamp, Vietnam | This study |
| *Coccomyxa subellipsoidea* E. Acton  (strain VP336) | Solitary, ellipsoidal, pointed on one side, sometimes slightly curved, asymmetrical, without mucilaginous sheath | 5.0–7.1 × 1.6–3.9 | Parietal, trough-shaped, thin, straight or with small concavity in the middle, covering about half of the cell volume | 2–4 autospores | Soil, tropic forest, Vietnam | This study |
| *Coccomyxa parasitica* R.N. Stevenson et G.R. South | Very variable in shape from spherical to elliptical, oblong or sickle-shaped, frequently with an attenuated hyaline tip | 1–11 x n/d | Parietal, usually single, or up to three present, often cup-shaped. | 2, 4, 8 (16) autospores | Parasitic, in globular, irregular or coalesced colonies in the mantle, muscle, gonad, gills or inner shell of *Placopecten magellanicus* Gmelin | Stevenson and South, 1974 |
| *Coccomyxa onubensis* Garbayo et al. ex J.L Fuentes et al. | Solitary or occasionally assembled in star-like structures, elongated  ovoid, asymmetric | 5.8–8.0 × 2.7–3.3 | Parietal, trough-shaped, covering  more than half of the cell volume Lipid droplets present in the cytosol. Starch grains present in the chloroplast | 2–4 autospores | Acidic waters,  Tinto River, Huelva, Andalucía, Southwestern Spain | Fuentes at al., 2016 |
| *Coccomyxa polymorpha* Darienko et Pröschold | Solitary or gathered in star-like structures, elongated-oval, asymmetric, very often curved. | 8.0–13.0 × 2.0–3.5 | Parietal, trough-shaped, thin with small concavity in the middle where the nucleus  is located. | 2–4 autospores | Unknown | Darienko et al., 2015 |
| *Coccomyxa vinatzeri* Darienko et Pröschold | Elongated oval, occasionally slightly pointed on one side | 4.0–6.2 × 1.9–3.5 | Parietal trough-shaped,  massive, covering more than half of the cell volume | 2–4 autospores, protoplast division is oblique and rarely traverse | Soil, Italy | Darienko et al., 2015 |
| *Coccomyxa galuniae* Darienko et Pröschold | Assembled in star-like structures, elongated-ovoid, straight or slightly dorsiventral, with the mucilage cap on the narrow end of the cell (or sometimes on both). | 7.0–10.5 × 2.3–4.0 | Parietal, trough-shaped without incisions | 2–4 autospores, always by oblique division of protoplast. | Freshwater and soil, Germany | Darienko et al., 2015 |
| *Coccomyxa* *melkonianii* V. Malavasi et P. Škaloud | Narrowly ellipsoidal and slightly asymmetrical, regularly curved, with rounded apices and without mucilaginous sheath | 6.0–8.5 × 3.0–5.4 | Parietal, cup-shaped, covering  much of the inner cell wall surface, with starch granules within the interthylacoidal spaces | 2–4 autospores | Freshwater, Rio Irvi River with a high content of heavy metals (Cd, Co, Fe, Mn and Zn), pH 6.85,  Sardinia, Italy | Malavasi et al., 2016 |
| *Coccomyxa*  *actinabiotis* Rivasseau, Farhi et Coute | Solitary, ellipsoidal, elongated-ovoid | 5.9–7.8 × 3.2–4.0 | Parietal with starch | 2–4 autospores | Freshwater pool used to cool the spent nuclear fuel elements of a nuclear research reactor, pH 5.1–5.4 | Rivasseau et al., 2016 |
| *Coccomyxa silvae‑gabretae* Barcytė et Nedbalová | Solitary, ellipsoidal, often elongated-oval, highly variable in length. | 4.1–8.2 × 2.0–3.3 | Parietal trough-shaped, occupying half of the cell volume | 2–4 autospores | Freshwater, acidified lake, pH ~ 5, Czech Republic | Barcytė and Nedbalová, 2017 |
| *Coccomyxa fottii* Barcytė et Nedbalová | Solitary, elongated ellipsoidal, both ends are rounded with one apex often slightly narrower | 5.4–9.7 × 2.0–3.4 | Parietal is trough-shaped, massive, and without pyrenoid.  Lipid droplets are present in the protoplast. | 2–4 autospores | Freshwater, thermal spring, Slovakia | Barcytė and Nedbalová, 2017 |
| *Coccomyxa greatwallensis* S. Cao et Q. Zhou | Ovoid to long ellipsoidal, asymmetrical, without mucilaginous sheath | 6.0–12.0 × 3.0–5.0 | Parietal, without pyrenoid and with starch granules in the inter thylacoidal spaces. Protoplast filled with lipid droplets | Not observed | Epiphytic, living with lichen *Psoroma hypnorum* (Vahl) Gray, on soil, Fildes Peninsula, Antarctica | Cao et al., 2018 |
| *Coccomyxa antarctica* S. Cao et Q. Zhou | Ovoid to ellipsoidal, asymmetrical | 8.0–12.0 × 4.0–7.0 | Parietal, with starch granules  in interthylacoidal spaces | Not observed | Epiphytic, living with lichen *Usnea aurantiacoatra* (Jacq.) Bory, on stone, Fildes Peninsula, Antarctica | Cao et al., 2018 |
| *Coccomyxa cimbrica* K. Sciuto, B. Baldan et I. Moro | Ellipsoidal to ovoid, variable in size, occasionally with small mucilaginous caps. | 4.5–7.0 × 2.0–3.0 | Parietal, single, cup-shaped | 2–4 autospores | Freshwater, peat bog, Italy | Sciuto et al., 2019 |

**References**

1. Barcytė, D., and Nedbalová, L. (2017). *Coccomyxa*: a dominant planktic alga in two acid lakes of different origin. *Extremophiles* 21, 245–257. doi: 10.1007/s00792-016-0899-6
2. Cao, S., Zhang, F., Zheng, H., Peng, F., Liu, C., and Zhou, Q. (2018). *Coccomyxa greatwallensis* sp. nov. (Trebouxiophyceae, Chlorophyta), a lichen epiphytic alga from Fildes Peninsula, Antarctica. *PhytoKeys* 110:39. doi: 10.3897%2Fphytokeys.110.26961
3. Darienko, T., Gustavs, L., Eggert, A., Wolf, W., and Pröschold, T. (2015). Evaluating the species boundaries of green microalgae (*Coccomyxa*, Trebouxiophyceae, Chlorophyta) using integrative taxonomy and DNA barcoding with further implications for the species identification in environmental samples. *PLOS* *ONE* 10(6):e0127838. doi: 10.1371/journal.pone.0127838
4. Fuentes, J., Huss, V., Montero, Z., Torronteras, R., Cuaresma, M., Garbayo, I., and Vílchez, C. (2016). Phylogenetic characterization and morphological and physiological aspects of a novel acidotolerant and halotolerant microalga *Coccomyxa onubensis* sp. nov. (Chlorophyta, Trebouxiophyceae). *J*. *Appl*. *Phycol*. 28, 3269–3279. doi: 10.1007/s10811-016-0887-3
5. Malavasi, V., Škaloud, P., Rindi, F., Tempesta, S., Paoletti, M., and Pasqualetti, M. (2016). DNA-based taxonomy in ecologically versatile microalgae: a re-evaluation of the species concept within the coccoid green algal genus *Coccomyxa* (Trebouxiophyceae, Chlorophyta). *PLOS ONE* 11(3):e0151137. doi: 10.1371/journal.pone.0151137
6. Rivasseau, C., Farhi, E., Compagnon, E., de Gouvion Saint Cyr, D., van Lis, R., Falconet, D., Kuntz, M., Atteia, A., and Couté, A. (2016). *Coccomyxa actinabiotis* sp. nov. (Trebouxiophyceae, Chlorophyta), a new green microalga living in the spent fuel cooling pool of a nuclear reactor. *J*. *Phycol*. 52(5), 689–703. doi: 10.1111/jpy.12442
7. Sciuto, K., Baldan, B., Marcato, S., and Moro, I. (2019). *Coccomyxa cimbrica* sp. nov., a green microalga found in association with carnivorous plants of the genus *Drosera* L. *Eur*. *J*. *Phycol*. 54(4), 531–547. doi: 10.1080/09670262.2019.1618920
8. Stevenson, R. N., and South, G. R. (1974). *Coccomyxa parasitica* sp. nov. (Coccomyxaceae, Chlorococcales), a parasite of giant scallops in Newfoundland. *Br*. *Phycol*. *J*. 9(3), 319–329. doi: 10.1080/00071617400650391
