# Supplementary material for: Phylogeny and fatty acid profiles of new Coccomyxa (Chlorophyta) species from soils of Vietnam
Source: Front Microbiol. 2025 Jul 14;16:1517865. doi: 10.3389/fmicb.2025.1517865 (PMC12301341; doi:10.3389/fmicb.2025.1517865)
Supplement: Supplementary file 3 [file Table_3.docx]

SUPPLEMENTARY MATERIAL 3

TABLE 1 Percent similarity (*p*-distance) matrix of 10 strains based on the 18S rDNA–ITS1–5.8S rDNA region (2,203 bp).

|  | Strain | 1 | 2 | 3 | 4 | 5 | 6 | 7 | 8 | 9 | 10 |
| --- | --- | --- | --- | --- | --- | --- | --- | --- | --- | --- | --- |
| 1 | *C*. *subellipsoidea* VP336 PV748123 | – |  |  |  |  |  |  |  |  |  |
| 2 | *C*. *fusiformis* VP339 PV748126 | 98.7 | – |  |  |  |  |  |  |  |  |
| 3 | *C*. *subellipsoidea* NIES 2252 HG972973 | 99.0 | 98.8 | – |  |  |  |  |  |  |  |
| 4 | *C*. *simplex* CCAP 812/3 HG972972 | 99.0 | 98.8 | 99.7 | – |  |  |  |  |  |  |
| 5 | *C*. *subellipsoidea* SAG 216-13 HG972978 | 99.3 | 99.0 | 98.9 | 98.9 | – |  |  |  |  |  |
| 6 | *C*. *subellipsoidea* SAG 69.80 HG972977 | 99.2 | 98.9 | 98.8 | 98.8 | 99.9 | – |  |  |  |  |
| 7 | *C*. *subellipsoidea* KN-2011-C13 HE586510 | 99.3 | 99.1 | 98.9 | 98.9 | 99.9 | 99.8 | – |  |  |  |
| 8 | “*Choricystis* sp.” CAUP H5105 HG972974 | 99.2 | 98.9 | 98.7 | 98.8 | 99.5 | 99.4 | 99.4 | – |  |  |
| 9 | *C*. *simplex* SAG 216-9a FN298926 | 97.6 | 97.1 | 97.5 | 97.6 | 97.7 | 97.7 | 97.7 | 97.6 | – |  |
| 10 | *C*. *polymorpha* CAUP H5101 HG972979 | 94.9 | 94.5 | 94.6 | 94.7 | 94.7 | 94.7 | 94.6 | 94.7 | 95.0 | – |

TABLE 2 Percent similarity (*p*-distance) matrix of 11 strains based on the 18S rDNA–ITS1–5.8S rDNA region (2,227 bp).

|  | Strain | 1 | 2 | 3 | 4 | 5 | 6 | 7 | 8 | 9 | 10 | 11 |
| --- | --- | --- | --- | --- | --- | --- | --- | --- | --- | --- | --- | --- |
| 1 | *C*. *tropica* VP521 PV748127 | – |  |  |  |  |  |  |  |  |  |  |
| 2 | *Coccomyxa* sp. KN-2011-T3 HE586515 | 100 | – |  |  |  |  |  |  |  |  |  |
| 3 | *Coccomyxa* sp. LH08AW1017 KP081392 | 100 | 99.9 | – |  |  |  |  |  |  |  |  |
| 4 | “*C*. *onubensis*” ACCV1 HE617183 | 98.4 | 98.5 | 99.8 | – |  |  |  |  |  |  |  |
| 5 | “*C*. *actinabiotis*” KN-2011-T4 HE586516 | 98.4 | 98.3 | 99.8 | 97.5 | – |  |  |  |  |  |  |
| 6 | “*C*. *actinabiotis*” CCAP 216/25 FR850476 | 98.5 | 98.5 | 99.9 | 97.6 | 99,8 | – |  |  |  |  |  |
| 7 | *C*. *polymorpha* CAUP H5101 HG972979 | 97.0 | 97.3 | 99.9 | 96.6 | 97.0 | 97.2 | – |  |  |  |  |
| 8 | *C*. *polymorpha* KN-2011-T2 HE586514 | 98.0 | 98.2 | 99.9 | 97.5 | 97.9 | 98.1 | 98.9 | – |  |  |  |
| 9 | *C*. *vinatzeri* ASIB V16 HG972994 | 94.6 | 94.5 | 98.1 | 94.3 | 93.8 | 94.0 | 93.7 | 94.1 | – |  |  |
| 10 | *C*. *galuniae* CCAP 211/97 FN298928 | 95.1 | 95.0 | 98.2 | 94.7 | 94.2 | 94.4 | 93.9 | 94.3 | 97.1 | – |  |
| 11 | *C*. *subellipsoidea* SAG 216-13 HG972978 | 95.5 | 95.6 | 98.6 | 95.6 | 94.9 | 95.1 | 94.6 | 94.9 | 95.9 | 95.9 | – |

TABLE 3 Percent similarity (*p*-distance) matrix of 13 strains based on the 18S rRNA gene (1,788 bp).

|  | Strain | 1 | 2 | 3 | 4 | 5 | 6 | 7 | 8 | 9 | 10 | 11 | 12 | 13 |
| --- | --- | --- | --- | --- | --- | --- | --- | --- | --- | --- | --- | --- | --- | --- |
| 1 | *C*. *cattiensis* VP449 PV748124 | – |  |  |  |  |  |  |  |  |  |  |  |  |
| 2 | *C*. *cattiensis* VP451 PV748125 | 100 | – |  |  |  |  |  |  |  |  |  |  |  |
| 3 | *Coccomyxa* sp. CPCC 508 AM981206 | 100 | 100 | – |  |  |  |  |  |  |  |  |  |  |
| 4 | “*C*. *parasitica*” CP-01 LN879479 | 99.9 | 99.9 | 99.9 | – |  |  |  |  |  |  |  |  |  |
| 5 | “*C*. *parasitica*” Flensburg fjord 1 EU127470 | 99.6 | 99.6 | 99.7 | 99.6 | – |  |  |  |  |  |  |  |  |
| 6 | “*C*. *parasitica*” Flensburg fjord 2 EU127471 | 99.7 | 99.7 | 99.7 | 99.6 | 99.4 | – |  |  |  |  |  |  |  |
| 7 | *C*. *antarctica* AEW8R K237 KP081329 | 99.1 | 99.1 | 99.1 | 99.0 | 98.9 | 98.9 | – |  |  |  |  |  |  |
| 8 | *C*. *antarctica* FACHB-2140 MF465900 | 99.4 | 99.4 | 99.4 | 99.3 | 99.1 | 99.1 | 99.7 | – |  |  |  |  |  |
| 9 | *C*. *cimbrica* DCDR LS999578 | 99.3 | 99.4 | 99.3 | 99.2 | 98.9 | 99.0 | 99.0 | 99.4 | – |  |  |  |  |
| 10 | *C*. *viridis* SAG 216-14 HG973002 | 99.7 | 99.7 | 99.7 | 99.6 | 99.3 | 99.3 | 99.4 | 99.7 | 99.3 | – |  |  |  |
| 11 | *C*. *mucigena* SAG 216-4 HG973001 | 99.7 | 99.7 | 99.7 | 99.6 | 99.3 | 99.3 | 99.4 | 99.7 | 99.3 | 100 | – |  |  |
| 12 | *C*. *greatwallensis* FACHB-2139 MF465899 | 99.2 | 99.2 | 99.3 | 99.4 | 98.9 | 99.2 | 99.4 | 99.4 | 99.0 | 99.5 | 99.5 | – |  |
| 13 | *C*. *dispar* SAG 49.84 HG972998 | 97.9 | 97.9 | 97.9 | 97.7 | 97.5 | 97.5 | 97.4 | 97.7 | 97.6 | 97.7 | 97.7 | 97.3 | – |
